# Death anxiety and religiosity in a multicultural sample: a pilot study examining curvilinearity, age and gender in Singapore

**DOI:** 10.3389/fpsyg.2024.1398620

**Published:** 2024-05-22

**Authors:** Radiah Maria Belak, Kay Hee Goh

**Affiliations:** Singapore University of Social Sciences, Singapore, Singapore

**Keywords:** death anxiety, spirituality, religiosity, existentialism, death and dying, transdiagnostic construct

## Abstract

This study investigated the association between multidimensional death anxiety and religiosity in multicultural Singapore by examining potential variations by age and gender. We also explored the possibility of a curvilinear effect, where highly religious or non-religious individuals report lower death anxiety than moderately religious people, forming an inverted U-curve pattern. Data were collected from 110 participants using questionnaires that assessed death anxiety and religiosity. Parametric and non-parametric tests were then conducted. The findings showed that women had significantly higher death anxiety and religiosity than men, and highly and moderately religious people had significantly higher death anxiety than non-religious people. People of all age groups had similar levels of death anxiety. These findings highlight the importance of developing targeted death anxiety interventions that integrate spiritual aspects in Singapore so that clinicians can provide culturally competent care.

## Introduction

Death is a certain and inevitable event for every living thing, as famously stated in the idiom “Nothing is certain but death and taxes.” Despite numerous studies on the subject, the nature of death, with its absolute finality and anticipated oblivion, is elusive to mankind. From MacDougall’s (1907) early attempt to measure the weight of the human soul to modern-day scientific investigations of near-death experiences (see Cassol et al., 2020), the only finding is that what happens after death remains inscrutable. It is unsurprising then for some people to confront death prematurely in the form of death anxiety (DA)–a universal experience across many societies (Jong et al., 2018).

There are many definitions for DA. Some researchers have described it as a fear of one’s own dying process, including any potential pain that may be experienced, as well as a fear of others’ death and their dying process (Farley, 2004). From an existential perspective, DA can be seen as a fear of losing one’s ability to experience life and the cessation of the sense of self (McCarthy, 1980; Tomer and Eliason, 1996). Consequently, DA is considered a multidimensional concept that manifests differently in individuals, rather than a unidimensional construct measured by a single scale or dimension (Lester, 1990; Cai et al., 2017). In this study, we adopt a multidimensional perspective when measuring DA, which encompasses both the fear of one’s own death and dying process and the fear of others’ death and their dying process. This approach is chosen to accommodate the complexity of the concept and the diverse ways it is experienced, enabling a nuanced exploration of DA.

The definition of religion has been the subject of ongoing debate among scholars, with no standardized definition despite efforts by prominent sociologists such as Durkheim, Weber, and Marx (Launay, 2022). This is largely due to the subjective and personal nature of religion and the diversity of its expressions (Bruce, 2011). Adding to the complexity, the terms spirituality and religion have been used interchangeably in past research, including in the articles we reference in this study. Our investigation indicates that religion and spirituality share common characteristics (Nelson, 2009). Essentially, religion refers to “a search for significance in ways related to the sacred” (Pargament, 1997, p. 32), while spirituality refers to “a search for the sacred” (Pargament, 1999, p. 12). Within this framework, religion serves as a broader concept encompassing various aspects along the wide spectrum of spiritual practices, while spirituality represents a subset of religion that is focused on an individual’s personal quest for the sacred. We will use the umbrella term “religiosity,” which refers to the degree or extent of a person’s religious devotion, in this study to encompass variations in religious and spiritual beliefs within the multireligious context of Singapore.

**Figure 1:**
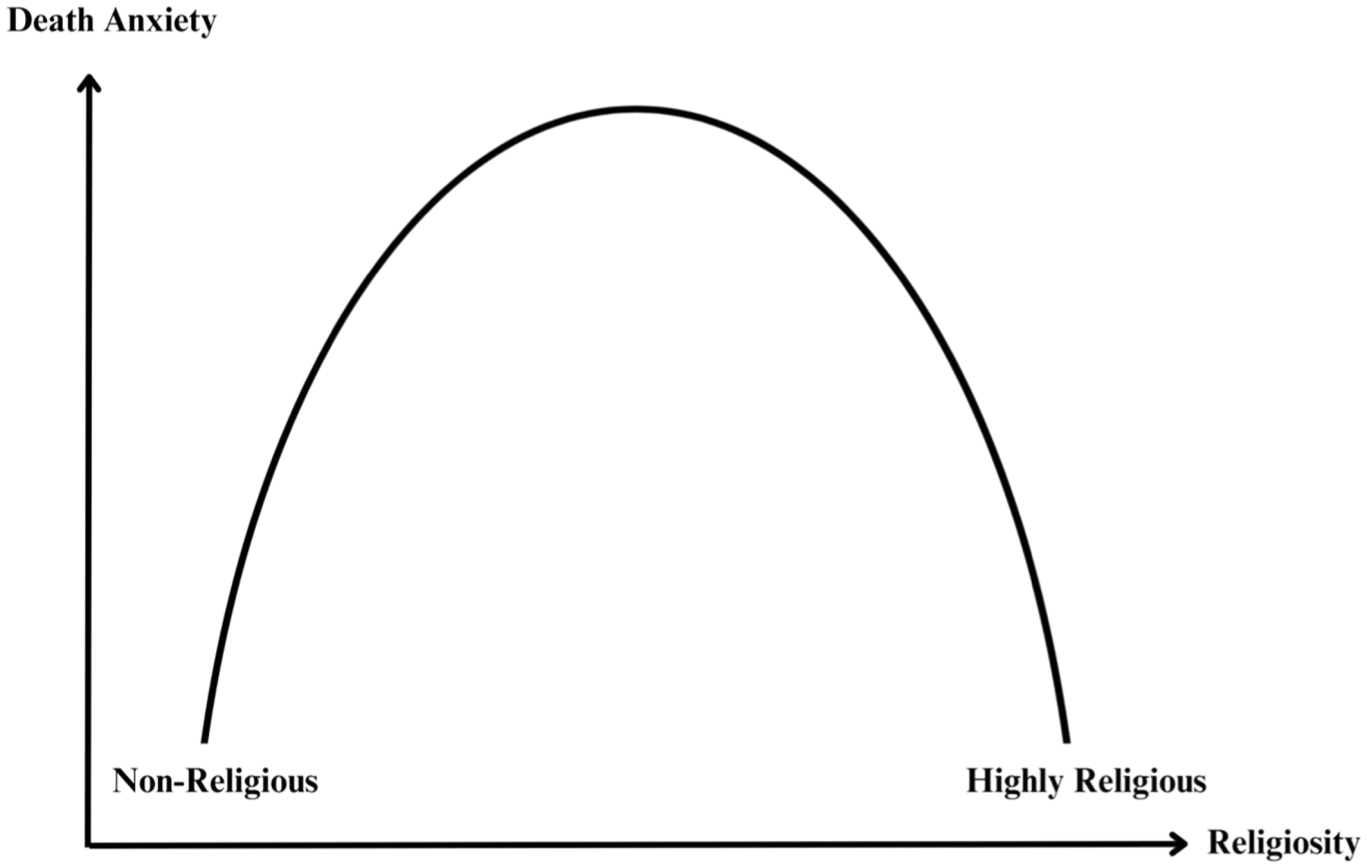
The inverted U-curve association between DA and religiosity.

Many individuals turn to religion as a coping mechanism for psychological distress, including DA (Friedrich-Killinger, 2020). Religion provides a source of meaning and serves as a valuable resource for managing personal challenges. However, the effectiveness of this coping strategy may vary depending on the intensity of an individual’s religious beliefs, as illustrated by the inverted U-curve pattern, hereafter referred to as the curvilinear relationship or curvilinear pattern (refer to Figure 1). In essence, this pattern describes a documented phenomenon in prior research where both low and high levels of religiosity are associated with lower levels of DA, while moderate levels of religiosity are linked to the highest levels of DA (Ellis and Wahab, 2013; Jong et al., 2018). The significance of this curvilinear relationship is its alignment with empirical evidence and existing theories concerning religiosity and DA, which will be discussed later. Accordingly, our research questions are as follows:

Does the relationship between DA and degree of religiosity follow a curvilinear pattern in a multicultural population such as Singapore? What is the relationship between DA, religiosity, age, and gender in this population?

## Literature review

### Problems

#### DA is a transdiagnostic construct

Foremost, this study’s significance lies in the evidence that DA is a transdiagnostic construct underlying numerous psychological disorders, meaning it is a shared, contributing, or perpetuating factor in many disorders such as depression, anxiety, somatic disorders, and personality disorders, amongst others (Iverach et al., 2014; Menzies et al., 2019). Failing to address DA may result in psychopathological symptoms that persist, relapse, or present as comorbidities in other mental illnesses (Menzies et al., 2018). Moreover, people who do not meet the diagnostic criteria for a psychological disorder may experience DA when dealing with low self-esteem, loneliness and major life changes such as aging, bereavement, or serious illness, which may potentially cause a range of maladaptive cognitive, emotional and behavioral responses (Sinoff, 2017; Zhang et al., 2019; Greenblatt-Kimron et al., 2021; Liu et al., 2022). Hence, further research in this field could inform the development of effective mental health interventions aimed at addressing the root cause of psychological disorders and reducing the mental health burden in Singapore.

#### Lack of religious sensitivity among clinicians

Mental health professionals often encounter individuals who struggle to come to terms with the concept of death (Yalom, 2008). Promisingly, research indicates that incorporating religious considerations into secular psychotherapy sessions can yield positive outcomes for those dealing with death-related anxieties (Post and Wade, 2009; Worthington et al., 2010; Friedrich-Killinger, 2020). Despite that, psychotherapy training programs often fall short of adequately integrating the exploration of religious matters within therapy (Post and Wade, 2009; Jafari, 2016). This shortfall may leave clinicians ill-prepared to effectively address the concerns of their religious clients. This significance is accentuated in Singapore, a nation where 80% of the population identifies as religious (Department of Statistics Singapore, 2021a). Addressing religious sensitivity among clinicians may thus be a pressing necessity, especially in a multicultural and spiritually diverse context like Singapore, where religiosity may function as a coping mechanism for individuals contending with DA.

#### Da-religiosity research gap in multicultural settings

Previous research on DA and religiosity has largely focused on Western or mono-religious contexts, such as those largely dominated by a single religion (Aday, 1985; Harding et al., 2005; Jong et al., 2018; Jung, 2018). Limited research has been conducted in polyreligious settings, making it challenging to generalize the findings to Singapore. According to the author’s knowledge, only one study has explored DA in Singapore and it involved palliative nurses instead of the general population (Tang et al., 2021). The scarcity of research in this domain may be attributed to the deeply rooted taboos surrounding death in Asian cultures, which often inhibit open conversations about mortality and related topics (Lee, 2009; Ang et al., 2016). This study seeks to address this gap by investigating the relationship between DA and religiosity in a diverse multicultural population such as Singapore and also by addressing the limitations of past research.

#### Mixed findings on DA and religiosity

Studies outside of Singapore have extensively examined the relationship between DA and religiosity, but the exact nature of this connection remains obscure (Ellis and Wahab, 2013; Jong et al., 2018). The most recent meta-analysis found highly heterogeneous findings (Jong et al., 2018). Of the 202 studies reviewed worldwide, half showed no statistically significant connection between DA and religiosity, 60 studies showed a negative correlation (higher religiosity linked with lower DA), 36 studies showed a positive correlation (higher religiosity linked to higher DA), and 10 studies supported a curvilinear relationship. The findings by Jong et al. (2018) echoed those of an earlier meta-analysis by Ellis and Wahab (2013), which also examined the relationship between religiosity and DA. The earlier meta-analysis found 32 studies with null findings, 40 studies with negative correlations, 27 studies with positive correlations, and 9 studies with curvilinear effects. It is evident that the relationship between DA and religiosity is multifaceted and warrants greater investigation.

#### Limitations in past studies on the curvilinear relationship

Prior research was predominantly fixated on linear effects, potentially neglecting the existence of curvilinear relationships (Ellis and Wahab, 2013; Jong et al., 2018). Consequently, Jong et al. (2018) identified support for the inverted U-curve association in only 10 studies, but most of these investigations exhibited significant methodological limitations. For example, McMordie (1981) excluded participants over the age of 44, a demographic crucial for understanding significant life transitions. In a similar vein, Downey (1984) confined their sample to middle-aged males. Conversely, Wink and Scott’s (2005) study solely featured elderly participants, potentially constraining the applicability of their findings to other age groups. Furthermore, more recent studies assessed by Jong et al. (2018) predominantly sampled college students.

Researchers from Jong et al.’s (2018) review of the 10 studies also underscored the need for future research to use multidimensional measures of religiosity and other age groups (Aday, 1985; Power and Smith, 2008; Wen, 2012). Indeed, most of the studies reviewed by Jong et al. (2018) on the curvilinear relationship used narrow definitions of DA and religiosity. Leming’s (1980) study overlooked the inherent multidimensionality of DA by measuring it as a unidimensional construct, which is inconsistent with the growing body of research suggesting that DA is a multidimensional construct (Lester, 1990; Iverach et al., 2014; Cai et al., 2017). Additionally, a significant number of studies have simplified religiosity into a binary classification of either religious or not, disregarding individuals who may fall somewhere in between on the religiosity spectrum (see Feldman et al., 2016; Cai et al., 2017; Liu et al., 2022).

The limitations of past research highlight the need to expand the research scope beyond a specific demographic, definition of religion, and definition of DA. Our study addresses this gap by including participants from three key age groups and using multidimensional measurements of religiosity and DA using valid and reliable scales. By doing so, we hope to obtain a more comprehensive and nuanced understanding of the DA-religiosity relationship, while also ensuring the inclusion of individuals in the middle of the religiosity spectrum, who may be particularly vulnerable to heightened levels of DA if the curvilinear relationship holds true in Singapore.

### Theories of DA and religiosity

#### Terror management theory

One of the most prominent theories of DA is terror management theory (TMT), which posits that one’s awareness of mortality is psychologically paralyzing, which motivates people to pursue literal or symbolic immortality to alleviate their fears (Greenberg et al., 2014). Literal immortality is defined as the conviction that one continues to exist after death, such as existing in heaven or being reincarnated, while symbolic immortality refers to enduring aspects of oneself, such as having offspring, cultural beliefs, and accomplishments that can convey lasting feelings of existence. Most religious beliefs offer both literal and symbolic immortality, the former through the promise of life continuing after death and the latter through membership in a lasting religious community and a sense of universal significance (Jong et al., 2018). Thus, as per TMT, individuals transcend DA through some form of immortality, whether literal, symbolic, or both.

When presented with stimuli that evoke mortality awareness, TMT posits that people respond by engaging in cultural and religious values defence, which involves favoring ingroups and denigrating outgroups to manage terror (Burke et al., 2010). This is the standard worldview defence component of TMT, which proposes that individuals alleviate DA by strengthening their worldviews and defending them against opposing views (Greenberg et al., 2014). According to Lifshin et al. (2021), individuals who adhere to the norms and values of their cultural or religious worldview may feel deserving of the ways in which that worldview offers to transcend death. In other words, defending one’s worldview leads to increased self-esteem, which correlates with a stronger conviction that one will persist symbolically or literally after death. Hence, the defence of cultural and religious values can serve as a coping mechanism for managing mortality awareness and ultimately alleviating DA.

TMT shares some commonalities with the curvilinear relationship. It suggests that individuals with strong religious or atheistic beliefs tend to experience lower levels of DA, while those with moderate beliefs may experience greater DA. According to TMT, this is because people with strong worldviews have a clear sense of either literal or symbolic immortality, which reduces their fear of death. This phenomenon is not limited to religious individuals, as even non-religious people with strong atheistic beliefs may find comfort in the prospect of death through their worldview’s provision of symbolic immortality as a defence mechanism (Jong et al., 2018). On the other hand, those with moderate religiosity may experience a reduction in their sense of literal or symbolic immortality, leading to higher levels of DA. The curvilinear relationship, as explained by TMT, highlights the complex interplay between the intensity of individuals’ religious worldviews and the level of DA.

#### Meaning management theory

Contrary to TMT, meaning management theory (MMT) posits that discovering meaningful, positive, and growth-oriented actions can mitigate DA (Wong, 2008). Based on this theory, humans construct and maintain shared meanings through social interaction, rather than simply reacting to objective events. MMT outlines two primary motivating forces in life: survival and the search for meaning behind survival (Wong, 2008). As a result, individuals are constantly engaged in constructing and reconstructing meaning, which helps them to make sense of the world around them. Religious belief can provide one way to discover meaning (Park, 2005). Therefore, Wong (2008) argues that the most effective way to alleviate DA is not through worldview defence, but rather through finding meaning in life and achieving self-actualization.

Religion provides a profound sense of meaning in life by providing a framework of beliefs, values and purpose that can help individuals navigate the complexities of the world and cope with personal challenges (Park, 2005). Religion offers meaning through either an extrinsic or intrinsic religious orientation (Li and Liu, 2023). Extrinsic religiosity refers to the participation in religious practices and beliefs primarily for instrumental reasons, such as social acceptance, tradition, or personal gains (Allport and Ross, 1967). Conversely, intrinsic religiosity refers to participation in religious practices primarily for internal reasons, such as personal growth and spiritual fulfilment (Allport and Ross, 1967). In short, an “extrinsically motivated person uses his religion, whereas the intrinsically motivated lives his religion” (Allport and Ross, 1967, p. 434). Research has demonstrated that extrinsic religiosity is linked to higher levels of DA and inferior mental health outcomes as compared to intrinsic religiosity (Power and McKinney, 2013; Jong et al., 2018). Although Wong (2008) emphasized the significance of establishing meaning to alleviate DA, it seems prudent to consider the type of meaning that is pursued, particularly regarding religious orientation.

A third religious orientation, Quest, was later introduced to account for the multidimensional nature of religiosity (Beck and Jessup, 2004). People with a Quest orientation are believed to be persistently searching for an ultimate truth that transcends their current understanding of religion (Batson et al., 1993). Their motivation is not necessarily to find final answers to their questions but rather to attain gratification in the process of seeking truth. This process encourages ongoing evaluation of spiritual beliefs, potentially leading to religious skepticism and doubt, which predicts DA (Batson et al., 1993; Henrie and Patrick, 2014). Furthermore, stigmatization from religious individuals toward those expressing doubt may deter them from seeking religious guidance, further cementing uncertainty and triggering DA (Upenieks, 2021). This aligns with the curvilinear relationship as individuals with a Quest religious orientation may be perceived as moderately religious individuals, and this group has been reported to have the highest DA.

#### Death apprehension theory

Death apprehension theory (DAT) offers a new perspective on DA, positing that as pain intensifies and enjoyment of life diminishes, death may become a less threatening alternative to continued living (Ellis et al., 2013). This has been supported by research (Cicirelli, 2003; Wysokiński et al., 2019). DAT also suggests that belief in literal immortality does not invariably reduce death apprehension, or DA. The degree of comfort provided by afterlife belief depends on factors such as the perceived difficulty of meeting religious standards, one’s adherence to those standards, and the belief that entrance to heaven or similar is contingent on following such standards (Ellis et al., 2013). In this view, highly religious individuals who diligently adhere to religious teachings may experience reduced DA, as they believe their strict adherence will earn them divine favor and, consequently, reduce the perceived risk of encountering adverse consequences in the afterlife.

DAT suggests that religious doubt can influence DA (Ellis et al., 2013), such that moderately religious individuals who doubt their ability to follow religious teachings may experience greater DA due to uncertainty about their afterlife fate or fear of punishment by a punitive God. Conversely, individuals who are confident in their ability to follow religious teachings may have lower levels of DA due to their belief in forgiveness and redemption. As per DAT, the relationship between religiosity and DA can be explained by the curvilinear relationship. This is because moderately religious individuals, who do not strictly adhere to God’s precepts, may experience elevated DA due to fears of punishment as compared to their highly religious counterparts. Thus, DAT suggests that belief in literal immortality may not be sufficient to reduce DA, and other factors related to religious belief and adherence must be considered. Ellis et al. (2013) suggested that further empirical investigation is required to explore DAT. Based on our literature review of the curvilinear relationship, TMT, MMT and DAT thus far, we have formed the following hypotheses:

*H*_0_: There is no significant difference in the mean DA scores of individuals across the three levels of religiosity.

*H*_1_: There will be a significant difference in mean DA scores across levels of religiosity, such that individuals who are highly religious or non-religious will report lower levels of DA compared to those who are moderately religious.

### Interactions between DA, religiosity, age and gender

#### Age, DA, and religiosity

##### Young and middle-aged adults

Existing literature shows that DA tends to be higher in young adulthood and middle age compared to later adulthood (Russac et al., 2007; Chopik, 2016). This phenomenon can be attributed to several factors. For young adults, the process of establishing their identity and psychologically separating themselves from their parents can amplify their awareness of mortality (McCarthy, 1980). Young adults who are in their child-bearing years may also be concerned about being able to raise children before dying or potentially leaving behind dependents in the event of a premature demise (Russac et al., 2007). For middle-aged adults, frequent experiences of losing loved ones such as parents or peers can remind them of the finality of life (Sinoff, 2017). Moreover, since both young and middle-aged adults tend to prioritize secular concerns over religious matters (Tornstam, 1997; Bengtson et al., 2015), this focus on worldly affairs may divert their attention from religiosity, which could otherwise serve as a coping mechanism for DA, thereby leaving their DA unmitigated.

##### Older adults

As people grow older, research shows they often experience decreased DA due to several factors. Life transitions associated with aging, such as declining health, increased isolation and shrinking social support networks, frequently lead older individuals to turn to religion (Idler, 2006; Krause, 2006, as cited in Bengtson et al., 2015). Additionally, older adults tend to have more free time to engage in religious activities compared to young individuals. This increased involvement contributes to the development of more extensive and refined religious coping mechanisms (Bengtson et al., 2015; Zimmer et al., 2016). Moreover, according to the gerotranscendence theory (Tornstam, 1997), older adults also tend to shift their focus from materialistic concerns to universal significance, placing greater value on relationships, personal growth, and community involvement. This shift in perspective can help older adults manage DA. Considering these factors, it is plausible to suggest that older adults may possess better religious coping mechanisms when dealing with DA than young and middle-aged adults.

##### Developmental patterns of DA

The patterns of DA across the lifespan discussed previously are consistent with Erikson’s (1980) stages of psychosocial development, which propose that individuals face and overcome different challenges at distinct stages of development. For example, both young and middle-aged adults may be preoccupied with intimacy and generativity issues, such as partnering, procreating, raising children and making a lasting impact on the world through secular involvements. Older adults, on the other hand, focus on transcendence or meaning through achieving a sense of integrity, which can lead to more accepting attitudes toward death. To explore whether these patterns are applicable in the unique context of Singapore regarding DA, we put forward the following hypotheses:

*H*_0_: There is no significant difference in mean DA scores across the three age groups.

*H*_2_: There will be a significant difference in mean DA scores across age groups, with young and middle-aged adults reporting higher levels of DA compared to older adults.

*H_0_*: There will be no significant difference in mean religiosity scores across young, middle-aged and older adults.

*H*_3_: There will be a significant difference in mean religiosity scores, with older adults reporting higher levels of religiosity than middle-aged and young adults.

#### Gender and religiosity

Extant literature suggests that women tend to be more religious than men (Francis and Penny, 2014; Hoffmann, 2019; Bryukhanov and Fedotenkov, 2023). However, the reasons for this gender difference remain debated. One theory, the risk preference theory, proposes that women’s greater religiosity may be due to their being more cautious and risk-averse than men, leading them to perceive irreligiosity as risky given the potential consequences of punishment in the afterlife (Miller and Hoffmann, 1995). However, recent studies have challenged this assumption, suggesting that personality traits may play a larger role in explaining gender differences in religiosity (Li et al., 2020; Vardy et al., 2022). For example, Penny et al. (2015) found that women tend to have lower levels of psychoticism than men. Psychoticism is associated with risk-taking, antisocial behavior, and impulsivity, and is a significant predictor of irreligiosity in men. Nevertheless, personality traits are not the focus of our study. Instead, we aim to address the need for broad measures of religiosity in understanding the relationship between religiosity and gender, as suggested by Ellis et al. (2013). Therefore, our hypotheses are:

*H*_0_: There is no significant difference in mean religiosity scores between men and women.

*H*_4_: There will be a significant difference in mean religiosity scores between men and women, with women reporting higher levels of religiosity compared to men.

#### Gender and DA

Despite their higher levels of religiosity, which is known to be a coping mechanism for dealing with DA, women in both Western and Asian populations still tend to experience greater DA than men (Ellis et al., 2013). This could be partially attributed to the higher levels of extrinsic religiosity among females (Flere, 2007). Saleem and Saleem (2019) suggest that in Pakistan, this difference may be linked to women’s limited freedom and lower social status, which can contribute to a reduced sense of symbolic immortality. Similar patterns have been observed in Iran, another conservative society characterized by traditional and restrictive gender roles (Adelirad et al., 2021).

In Western cultures, women may exhibit an external locus of control, believing that the negative consequences of aging are out of their control (Dattel and Neimeyer, 1990). This perception may lead to heightened concerns about dependence in later years, and, consequently, elevated DA. Furthermore, ageist stereotypes disproportionately affect women, who are frequently judged based on physical appearance and sexual desirability (Schafer and Shippee, 2010). Consequently, some women may view these attributes as sources of symbolic immortality, believing that physical appearance and attractiveness contribute to their enduring relevance, value, and remembrance. It is evident that cultural factors play a significant role in gender-related differences in DA. As such, our study explores gender differences and their association with DA in Singapore’s unique cultural context, paving the way for the following hypotheses:

*H*_0_: There is no significant difference in mean DA scores between men and women.

*H*_5_: There will be a significant difference in mean DA scores between men and women, with women reporting higher levels of DA compared to men.

## Methodology

### Reflexivity

Previous academic pursuits in media studies have exposed the researcher to global narratives detailing conflicts arising from religious and cultural disparities. These experiences have inspired the researcher to investigate the role of religious beliefs as a potential therapeutic resource. While acknowledging the value of methodological pluralism, the researcher has chosen a quantitative approach for this study to rigorously test hypotheses derived from the literature review.

### Philosophy

Our study relied on closed-ended online survey questions and statistical analyses, which align with the positivist paradigm’s emphasis on objective, quantifiable data for hypothesis testing and conclusion drawing (Park et al., 2020). The positivist philosophy serves as a fitting framework for our research, allowing for rigorous empirical testing of our research questions.

### Measures

The study utilized a cross-sectional design and data were collected through an online survey hosted on Qualtrics. Questions on consent and demographic data (age and gender) were first collected (see Appendix C). Age categories were as follows: young adults (ages 21 to 35), middle-aged adults (ages 35 to 64), and older adults (ages 65 and above). This aligns with the definitions provided by the Ministry of Culture, Community and Youth Singapore (2021) and the Department of Statistics Singapore (2021b), where youth is defined as individuals up to age 35, and the elderly category includes those aged 65 and above. Gender was categorized into three groups: male, female, and other. Following this, the survey contained two questionnaires: Lester’s (1990) Revised Collett-Lester Fear of Death Scale (CLFDS-R), consisting of 32 questions (refer to Appendix D), and the Centrality of Religiosity Scale (CRSi-7) developed by Huber and Huber (2012), which included seven questions (see Appendix E). Altogether, the survey comprises 41 items excluding the question on consent. Participants are only required to respond to 39 items with an anticipated completion time of approximately 15 min.

The CLFDS-R measures multidimensional DA using four subscales: “Your Own Death,” “Your Own Dying,” “Death of Others,” and “Dying of Others” (Lester, 1990). Each subscale uses a five-point Likert scale (1 = *not at all* to 5 = *very much*), with scores of 8 to 40 points. The overall scale score is calculated by averaging the subscale scores, resulting in a score of 32 to 160 points. Higher scores indicate greater DA. Scores should only be interpreted relative to others, as there are no predefined categories or absolute interpretations of CLFDS-R scores.

The CLFDS-R is a highly valid and reliable measure of DA that has been used across various populations (Tomás-Sábado et al., 2007; Venegas et al., 2011; Bužgová and Janíková, 2019; Cuniah et al., 2021). It has demonstrated robust internal consistency, test–retest reliability, construct validity and content validity. Most importantly, the CLFDS-R was selected for this study due to its distinctive multidimensional approach to assessing DA, which is rare among psychometrically sound scales in the field. A systematic review of available DA scales concluded that most existing scales have problematic properties (Zuccala et al., 2019). However, the researchers deemed the CLFDS-R to be the most favorable scale for clinical use due to its good responsiveness, which means it may detect meaningful changes in the variable it is designed to assess (Norman et al., 2007). This is particularly important for our study, which aims to lay the groundwork for effective clinical interventions for DA.

The CRSi-7 developed by Huber and Huber (2012) uses a five-point Likert scale (1 = *never* to 5 = *very often*). All items are combined to create a single index representing the mean score across all items, which ranges from 1 to 5. Based on the index score, participants are classified into one of three groups: non-religious (scores between 1.0 and 2.0), moderately religious (scores between 2.1 and 3.9) or highly religious (scores between 4.0 and 5.0). The CRSi-7 will assess participants’ degree of religiosity across five dimensions–ideology, intellect, religious experience, and private and public practice.

The CRSi-7, an abbreviated version of the original CRS, was chosen for its suitability in the diverse religious landscape of the Singapore population, which encompasses various Abrahamic and non-Abrahamic faiths, including Christianity, Islam, Hinduism, Buddhism, and more (Huber and Huber, 2012). It was also selected for its brevity, with the aim of minimizing respondent burden. More importantly, the CRSi-7 has demonstrated strong cross-cultural validity and reliability in both Asian contexts like the Philippines and Hong Kong, as well as in Western settings such as Russia, Georgia and Romania (Lee and Kuang, 2020; Ackert et al., 2020a,b; Ackert and Plopeanu, 2021; del Castillo et al., 2021). The scale exhibited consistently high internal consistency with Cronbach’s alphas values ranging from 0.67 to 0.93 across these studies, indicating good to excellent reliability. These values affirm the scale’s robustness in measuring the intended constructs effectively.

### Sampling strategy and demographics

Due to the sensitive nature of the study, we implemented strict inclusion criteria to ensure participants’ well-being. Participants had to be 21 years or older, Singaporean citizens or permanent residents, proficient in English, have no history of severe mental illness or cognitive impairments, and have no recent experiences of significant psychiatric symptoms, including but not limited to depression or suicidal ideation. We recruited participants using convenience sampling, advertising the study on social media (Facebook, WhatsApp, Telegram, Instagram, and Reddit) and distributing flyers at a public walkway beside Tampines Bus Interchange, an area with high foot traffic.

A total of 146 participants attempted the survey, but 36 dropped out halfway or did not complete it, resulting in an attrition rate of 25%. The demographics of the sample were as follows: 51 young adults (46.4%), 45 middle-aged adults (40.9%), and 14 older adults (12.7%). Additionally, 54 participants were female (49.1%), and 56 were male (50.9%). There were 16 non-religious participants (14.5%), 56 moderately religious participants (50.9%), and 38 highly religious participants (34.5%).

### Data analysis

This study assessed four variables: DA, religiosity, age, and gender, with the latter two being covariates. Numerical values were assigned to each response item for every question, and these values are subsequently used for analysis. DA was measured as a continuous numeric variable on the CLFDS-R scale. We calculated DA scores for each subscale, combined them, and determined the aggregate score. Religiosity scores for participants were computed by averaging their responses to all items, resulting in an index score.

Statistical analysis began with assessing data normality using the Shapiro–Wilk test, followed by a combination of parametric and non-parametric tests to test various hypotheses. Parametric tests, such as one-way ANOVA and independent samples *t*-test, were used for variables that exhibited normal distributions. Non-parametric tests, such as Kruskal-Wallis and chi-square tests, were employed for variables that did not conform to normality assumptions. All analyses took place on Statistical Package for the Social Sciences (SPSS).

### Procedure and ethical considerations

Measures were implemented to ensure adherence to ethical standards throughout the study. The study received approval from the Institutional Review Board. Before attempting the 41-item online survey, participants received a Participant Information Sheet (PIS) that outlines the study’s purpose, potential benefits, inclusion criteria, voluntary participation, right to withdraw, the confidentiality of responses, and researcher contact information (see Appendix A). Notably, the PIS states that the study includes potentially emotionally distressing questions, particularly those related to DA. To address this, the PIS provides support resources, including contact details for mental health professionals and helplines. Participants were also encouraged to take breaks or stop the questionnaire if they feel upset, with the option to cease participating at any time without incurring a penalty. Participants then had the option to consent or decline to participate in the informed consent form within the PIS (see Appendix B). After completing the survey, participants would see a debriefing statement on the screen summarizing the study’s purpose, procedures, and data handling practices (see Appendix F). They were again offered support resources and the research team’s contact information for any further questions or concerns.

## Results

### Assessing data normality

To ensure our data complies with the normality assumptions necessary for parametric tests, we conducted a normality assessment using the Shapiro–Wilk test. This test is suitable for our sample size of 110 since it is designed for use with sample sizes ranging from three to 5,000 (Yap and Sim, 2011). In the Shapiro–Wilk test, the null hypothesis assumes that the data adheres to a normal distribution, while the alternative hypothesis indicates a departure from normality. If the *p*-value falls below the chosen significance level of 0.05, we reject the null hypothesis. Conversely, if the *p*-value exceeds this threshold, we fail to reject the null hypothesis, indicating that the data follows a normal distribution.

**Figure 2:**
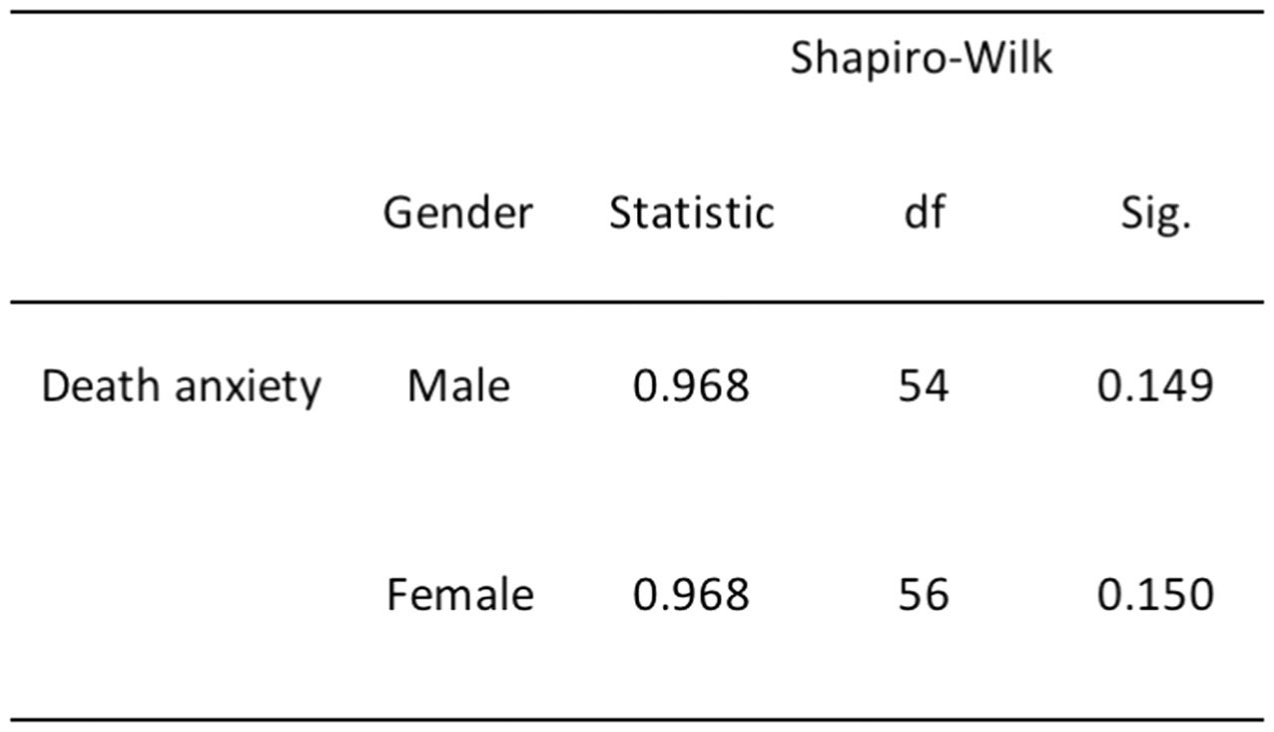
Normality test results for DA and gender.

**Figure 3:**
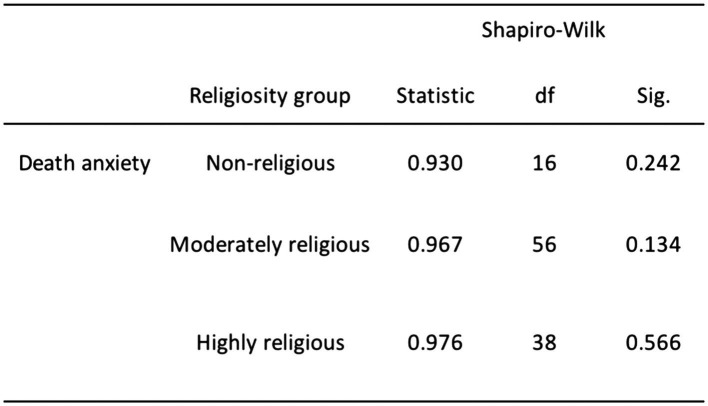
Normality test results for DA and religiosity.

Based on the results of the normality tests (as illustrated in Figure 2), we failed to reject the null hypothesis for DA with respect to both females (*p* = 0.149) and males (*p* = 0.150). Similarly, we failed to reject the null hypothesis for DA concerning religiosity (see Figure 3), including non-religious (*p* = 0.242), moderately religious (*p* = 0.134), and highly religious individuals (*p* = 0.566). These analyses revealed that our data distribution, specifically DA in relation to gender and religiosity, followed a normal distribution. Hence, we opted to run parametric tests such as the one-way ANOVA and independent sampled *t*-tests for these variables in our analysis.

**Figure 4:**
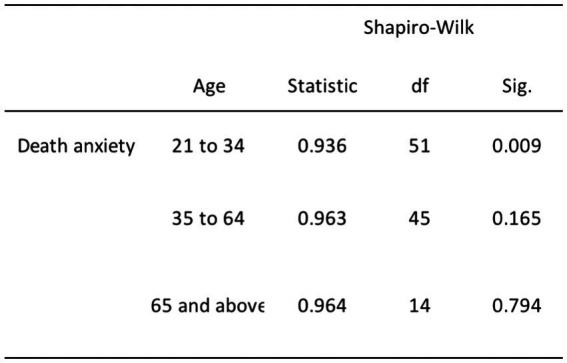
Normality test results for DA and age.

**Figure 5:**
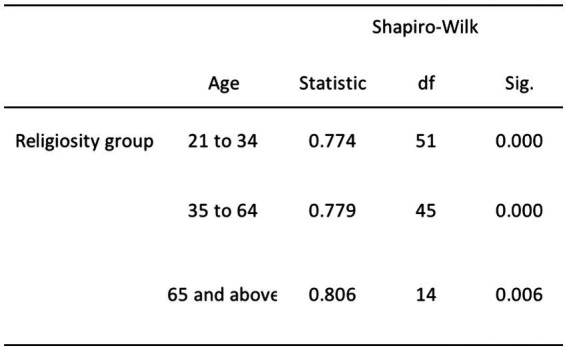
Normality test results for religiosity and age.

**Figure 6:**
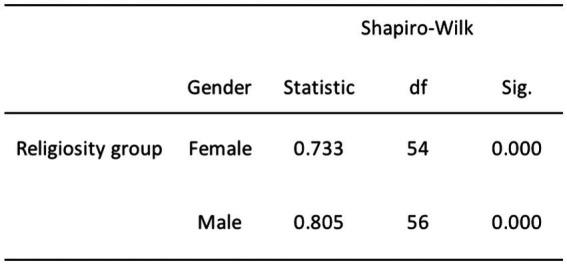
Normality test results for religiosity and gender.

The remaining relationships, which involve DA and age, religiosity and age, and religiosity and gender, exhibited departures from normality. Specifically, we rejected the null hypothesis for DA and age due to the non-normal distribution of the young adult age group (*p* = 0.009; refer to Figure 4). Furthermore, we rejected the null hypothesis for the relationship between religiosity and age, including both young and middle-aged adults (*p* < 0.001), as well as older adults (*p* = 0.006; see Figure 5). Additionally, we rejected the null hypothesis for the relationship between religiosity and both genders (*p* < 0.001; see Figure 6). Given the presence of statistically significant *p*-values, indicating deviations from normality, we chose to employ non-parametric tests, such as the chi-square and Kruskal-Wallis tests. These non-parametric tests are better suited for data that do not conform to the assumptions of parametric tests, enabling us to analyze the data effectively while accounting for the observed departures from normality (Yap and Sim, 2011; Gravetter and Wallnau, 2015).

### Hypotheses testing

**Figure 7:**
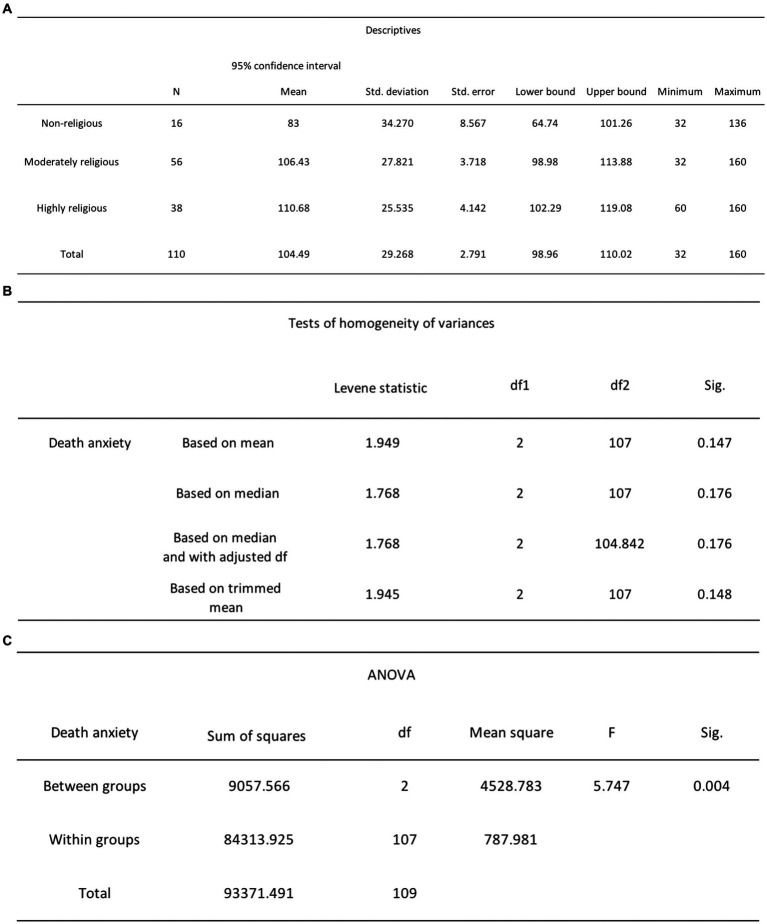
**(A)** One-way ANOVA descriptive statistics for DA and religiosity. **(B)** One-way ANOVA Levene’s test results for DA and religiosity. **(C)** One-way ANOVA test results for DA and religiosity.

**Figure 8:**
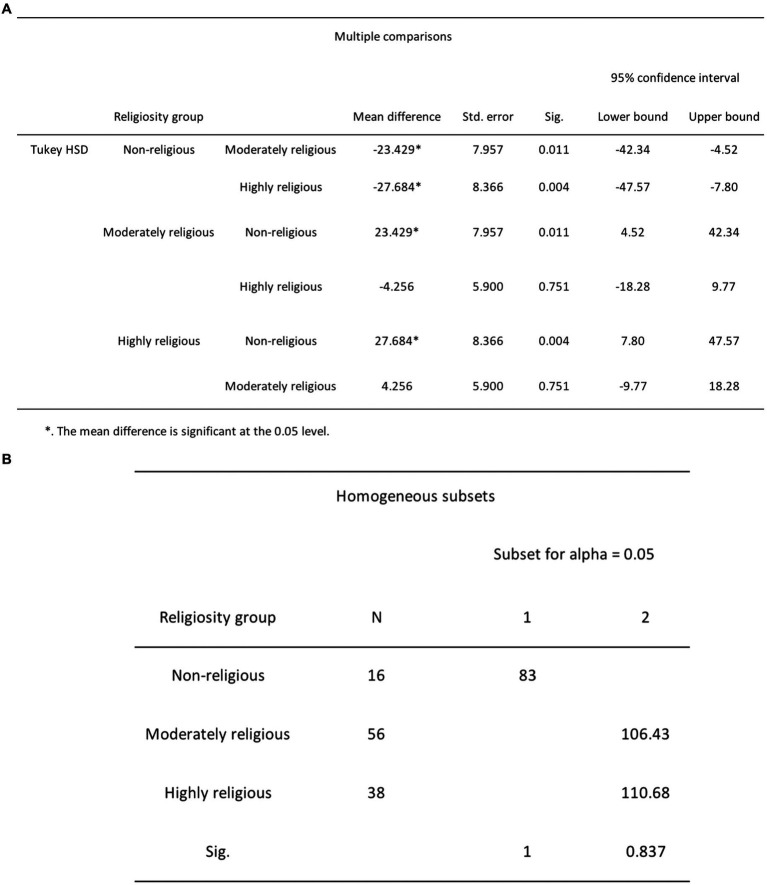
**(A)** Post-hoc Tukey HSD multiple comparisons test results for DA and religiosity. **(B)** Post-hoc Tukey HSD homogeneous subsets test results for DA and religiosity.

In *H*_1_, it was posited that highly and non-religious participants would report lower levels of DA scores compared to moderately religious participants. To test this hypothesis, we performed a one-way ANOVA, examining participants’ DA scores (the dependent variable) in relation to their degree of religiosity (the independent variable) with a significance level set at 0.05. The results showed a significant difference between the two variables *F*(2, 107) = 5.75, *p* = 0.004 (see Figure 7). Tukey’s HSD *post hoc* tests revealed that non-religious participants (*n* = 16, *M* = 83, SD = 34.27) had significantly lower DA scores than moderately religious participants (*n* = 56, *M* = 106.43, SD = 27.82, *p* = 0.011) and highly religious participants (*n* = 38, *M* = 110.68, SD = 25.54, *p* = 0.004; refer to Figure 8). However, there was no statistically significant difference in DA scores between moderately religious and highly religious participants (*p* = 0.75). Therefore, we reject the null hypothesis, which states that there is no significant difference in mean DA scores across the three levels of religiosity.

**Figure 9:**
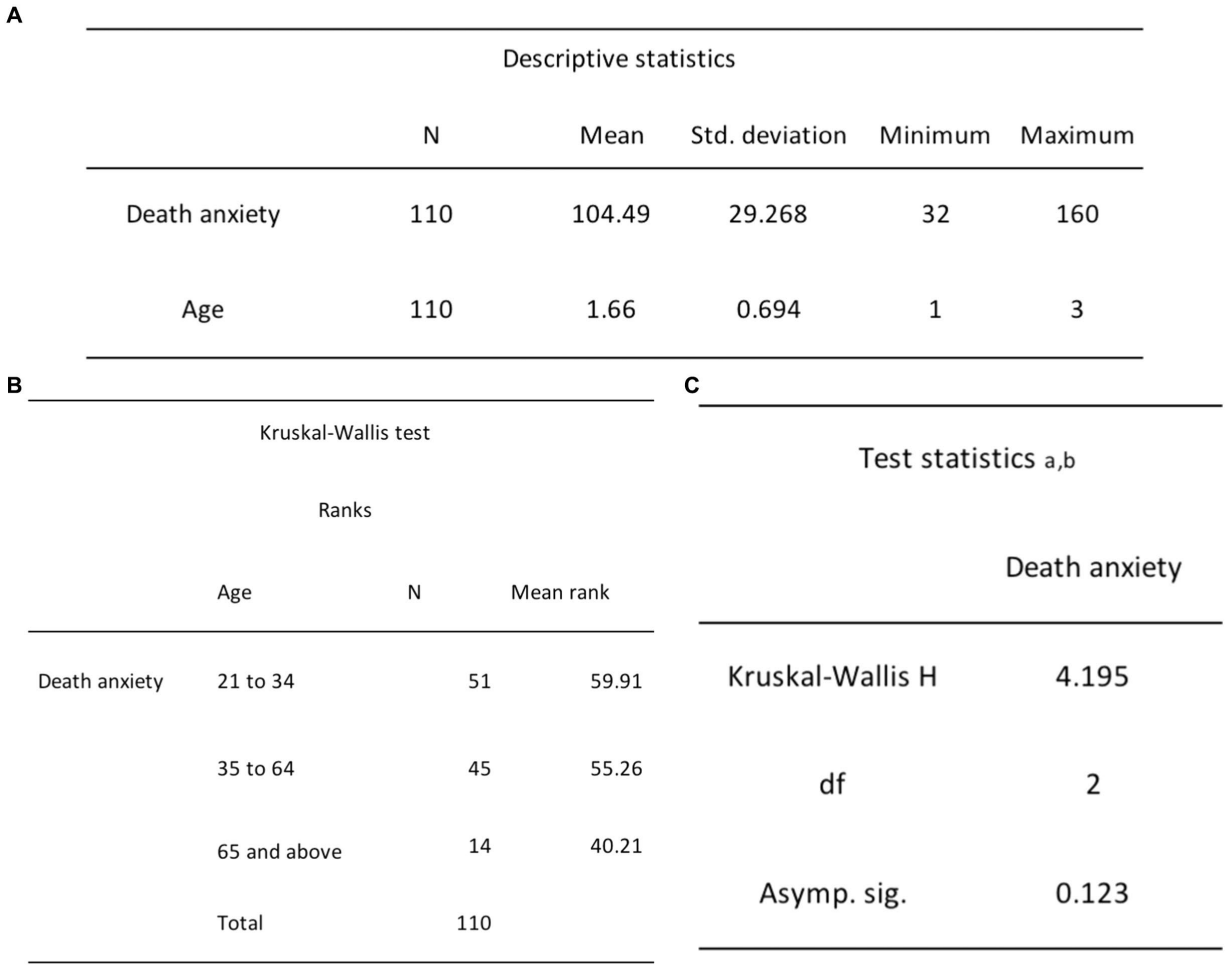
**(A)** Independent-samples Kruskal-Wallis descriptive statistics for DA and age. **(B)** Independent-samples Kruskal-Wallis ranks test results for DA and age. **(C)** Independent-samples Kruskal-Wallis test statistics for DA and age.

Subsequently, in *H*_2_, it was posited that young and middle-aged adults would exhibit higher DA scores compared to older adults. To test that, we conducted an independent-samples Kruskal-Wallis test on participants’ DA scores, the dependent variable, in relation to their age group, the independent variable. This analysis method was chosen as the Kruskal-Wallis test is a suitable and robust non-parametric alternative to ANOVA by enabling the assessment of differences between three or more independent groups with a continuous outcome variable (Vindras et al., 2012). The differences in rank totals, specifically 59.91 (young adults), 55.26 (middle-aged adults), and 40.21 (older adults), did not demonstrate statistical significance, as indicated by H (2, *n* = 110) = 4.2, *p* = 0.123 (see Figure 9). Based on these results, we fail to reject the null hypothesis, which states that there will be no significant difference in mean DA scores across the three age groups.

**Figure 10:**
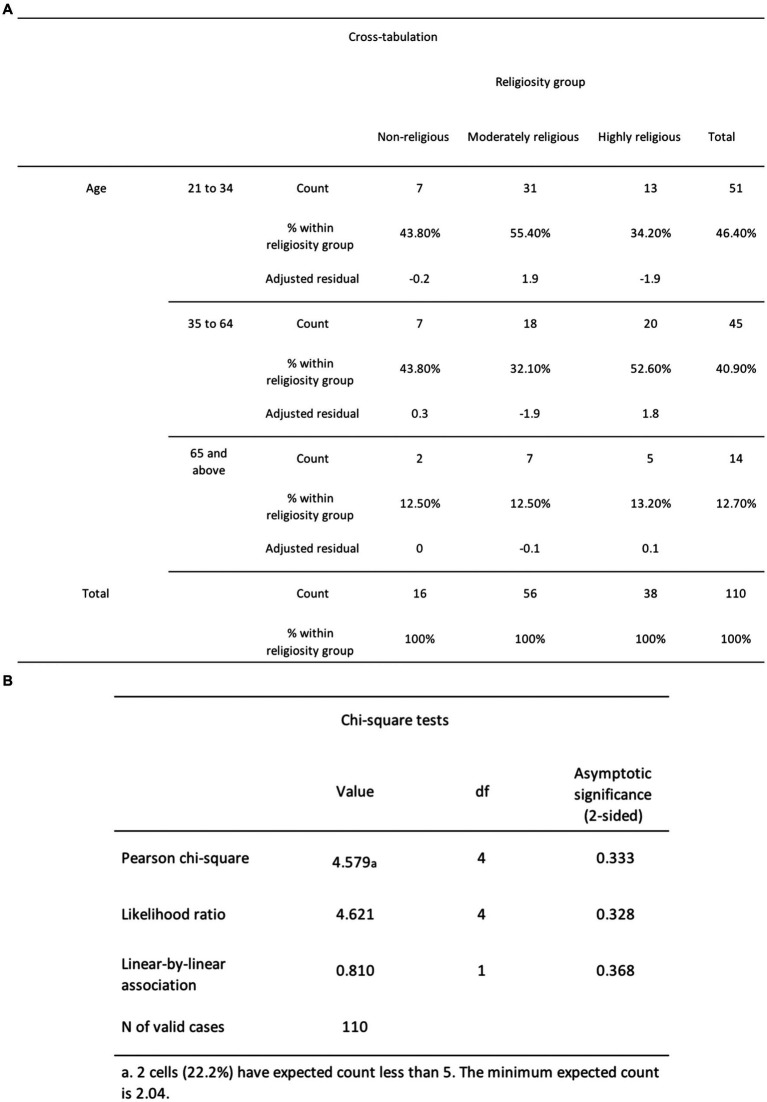
**(A)** The chi-square cross-tabulation test results for religiosity and age. **(B)** The chi-square test results for religiosity and age.

To test *H*_3_, which stated that older adults would report greater religiosity scores compared to middle-aged and young adults, we conducted the chi-square test of independence to assess the association between religiosity and age. We employed the chi-square test as it is a widely employed and reliable non-parametric method for examining the relationship between categorical variables (Gravetter and Wallnau, 2015). Our analysis revealed that the relationship between these variables was not statistically significant, as demonstrated by χ^2^ (4, *N* = 110) = 4.58, *p* = 0.333 (see Figure 10). Therefore, based on these findings, we fail to reject the null hypothesis, which states that there would be no significant difference in mean religiosity across the three age groups.

**Figure 11:**
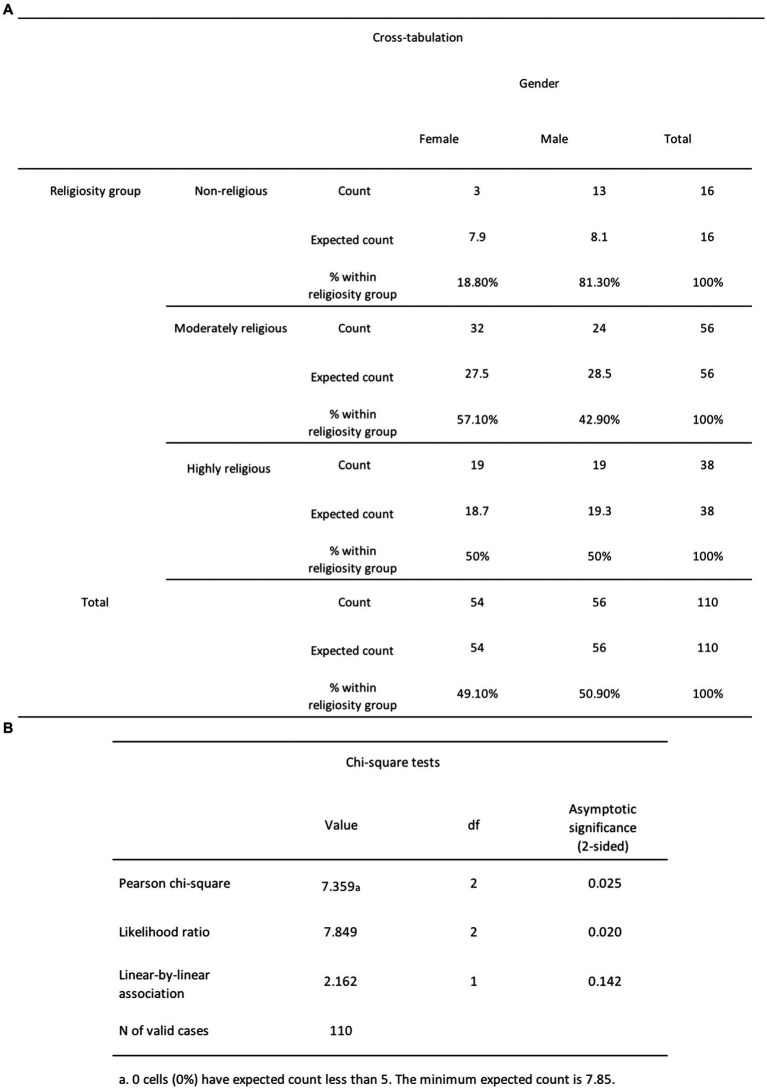
**(A)** The chi-square cross-tabulation test results for religiosity and gender. **(B)** The chi-square test results for religiosity and gender.

According to *H*_4_, women would report greater religiosity scores compared to men, so we conducted the chi-square test of independence to assess the association between DA and gender. The analysis revealed that the relationship between these variables was statistically significant (see Figure 11), as demonstrated by χ^2^ (2, *N* = 110) = 7.36, *p* = 0.025. The count for non-religious females (3) is lower than the expected count (7.9). The count for non-religious males (13) is higher than the expected count (8.1). The count for moderately religious females (32) is higher than the expected count (27.5). The count for moderately religious males (24) is lower than the expected count (28.5) The count for highly religious females (19) is higher than the expected count (18.7). The count for highly religious males (19) is lower than the expected count (19.3). From these findings, we reject the null hypothesis, which states that there is no significant difference in mean religiosity scores between men and women.

**Figure 12:**
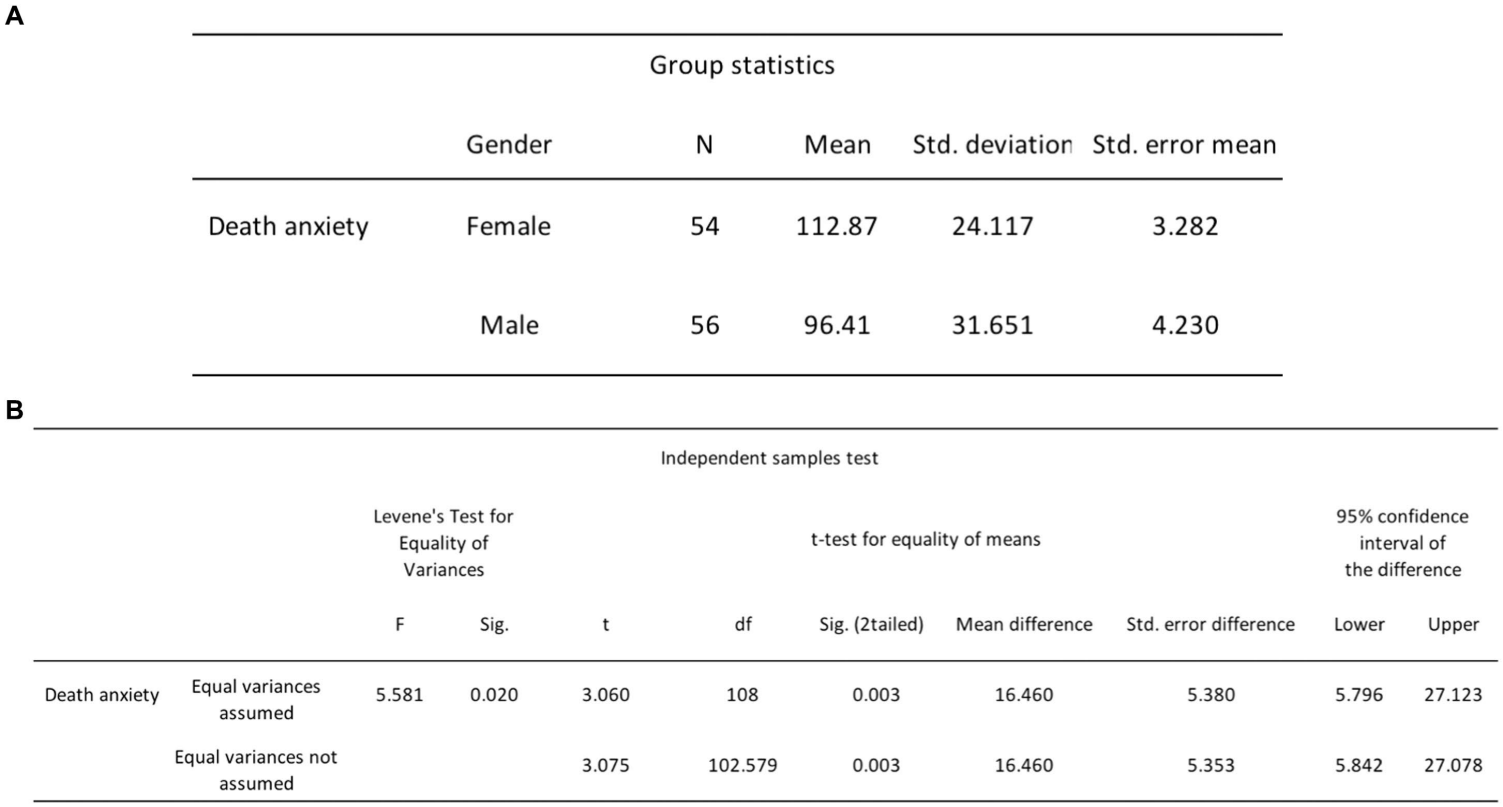
**(A)** Independent samples *t*-tests group statistics for DA and gender. **(B)** Independent samples *t*-tests results for DA and gender.

*H*_5_ posited that women would report greater DA scores compared to men. To test this, an independent samples *t*-test was performed to evaluate whether there was a difference between the DA scores of females and males. The results indicated that females (*M* = 112.87, SD = 24.12) reported significantly higher levels of DA than males (*M* = 96.41, SD = 31.65), *t*(108) = 3.06, *p* = 0.003 (see Figure 12). Thus, we reject the null hypothesis which states that there is no significant difference in mean DA scores between men and women.

## Discussion

### Shift from inverted U-curve to rising curve

We rejected the null hypothesis for *H*_1_, which assumed no significant difference in mean DA scores of individuals across the three levels of religiosity. Our findings challenged our initial expectations regarding the curvilinear relationship between religiosity and DA. It was anticipated that non-religious and highly religious individuals would exhibit reduced levels of DA, while moderately religious individuals would experience higher levels of DA. Contrary to this expectation, our findings unveiled a contrasting pattern–both highly and moderately religious participants reported higher levels of DA, while non-religious participants displayed the lowest levels of DA. Instead of the anticipated inverted U-curve pattern, our results indicated a rising curve, wherein a higher degree of religiosity was associated with greater levels of DA. This outcome contradicted our initial assumption that religiosity serves as a coping mechanism for DA, an idea supported by several past studies (Ellis and Wahab, 2013; Jong et al., 2018).

There are a few possible explanations for this unanticipated finding. One plausible explanation is that our study sample might be skewed toward a higher proportion of highly and moderately religious participants when compared to the number of non-religious individuals. Consequently, the greater instances of DA reported by these two majority groups could potentially explain the overall trend observed. However, it is crucial to emphasize that approximately 85% of our study sample identified as religious, a percentage that closely mirrors the population demographics of Singapore, where approximately 80% of the population identifies as religious. Therefore, our study sample can be viewed as a representative reflection of the general population, enhancing the external validity of our findings.

### The multifaceted nature of religiosity

Another possibility is that, as mentioned previously, religiosity presents itself in different orientations–intrinsic, extrinsic and Quest (Beck and Jessup, 2004; Li and Liu, 2023). The categorical approach of the curvilinear relationship, distinguishing religiosity into low, moderate, or high levels, may miss the nuances of these orientations. While religiosity may serve as a coping mechanism for DA, this effect may be specific to intrinsic religiosity, which is characterized by a deep, personally fulfilling connection to one’s faith (Allport and Ross, 1967). Intrinsically religious individuals view their faith as an end in itself, emphasizing the spiritual and personal aspects of their beliefs. Conversely, extrinsic religiosity, which is more directed toward using religion as a means to achieve personal goals or gain external rewards, may not offer the same level of alleviation against DA. This could stem from extrinsic religiosity being inherently transactional and utilitarian, with individuals leaning toward this orientation often engaging with religion for pragmatic purposes such as social acceptance or material gains, rather than pursuing profound spiritual fulfillment. As such, the instrumental nature of extrinsic religiosity may not offer the same level of solace and alleviation against DA that comes from a more profound and personally fulfilling connection with one’s faith.

Moreover, the concept of Quest religious orientation adds complexity to this relationship. Characterized by flexible and moderate religious beliefs, a Quest orientation may increase doubt toward one’s faith, which, according to the DAT, may heighten DA due to fear of afterlife punishment by a punitive God (Ellis et al., 2013; Henrie and Patrick, 2014). Singapore’s unique social landscape, a blend of multi-religiosity and secularism, suggests that a significant portion of the population may identify with Quest orientation. This assertion is supported by our sample, where approximately 51% of participants identify as moderately religious. Individuals within this category may share commonalities with Quest-oriented individuals. For instance, they may not strongly adhere to specific religious doctrines due to factors such as a belief in the impossibility of absolute certainty within a single faith, an ongoing search for ultimate truth, or exposure to diverse perspectives which can lead to greater tolerance and acceptance of different perspectives, potentially reducing the rigidity of religious beliefs.

**Figure 13:**
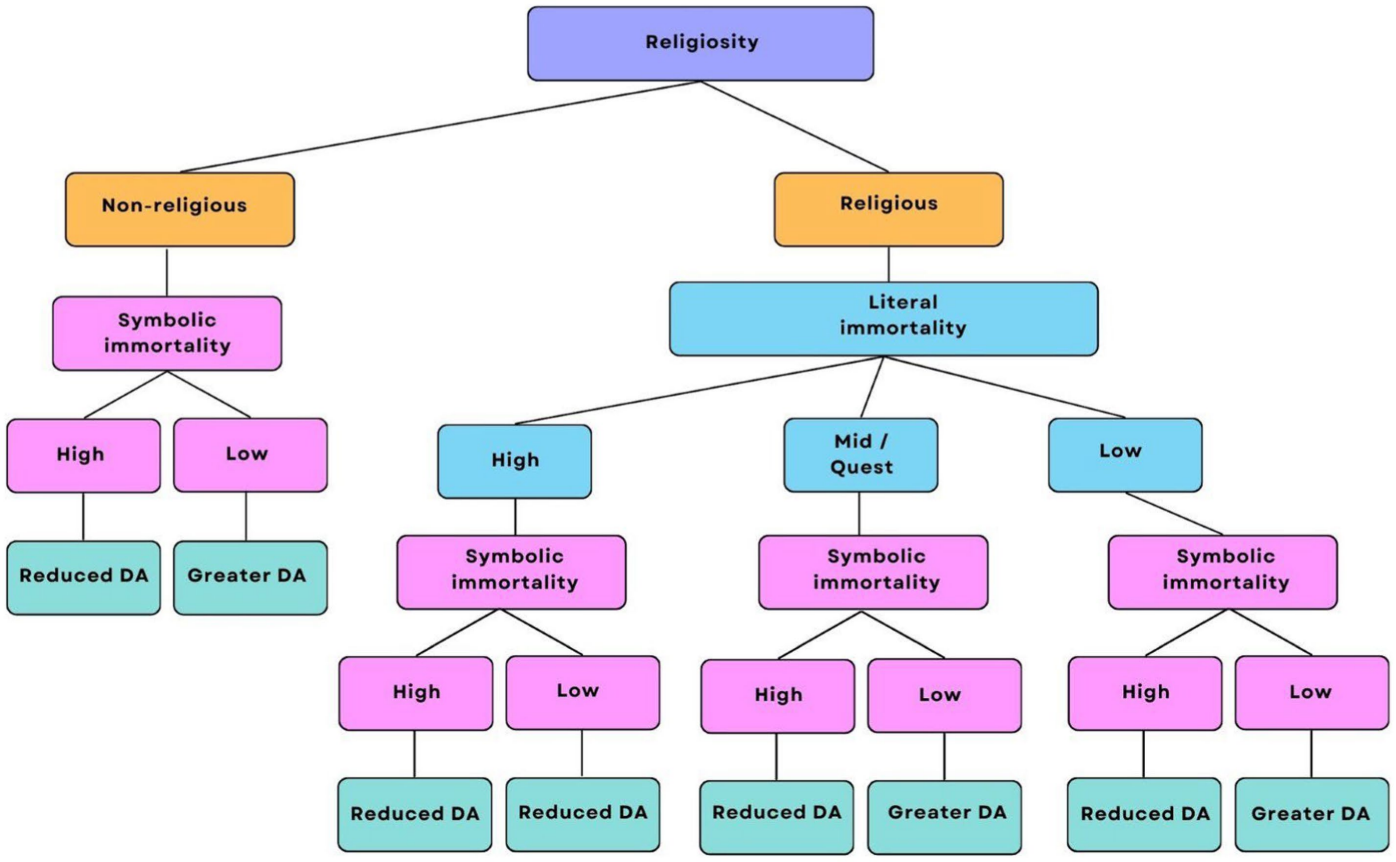
The relationship between religiosity and DA visualized in a tree diagram. This tree diagram is a visualization of the broad ways in which religiosity can be divided. It does not attempt to capture the full spectrum of religiosity, which may consist of various combinations of the factors in the diagram, nor is it an attempt to simplify religiosity into a linear process. Instead, it aims to provide a brief overview of the general trends of DA across different expressions of religiosity.

Besides religious orientation, the curvilinear relationship between religiosity and DA may also oversimplify the intricate nature of religiosity. It is imperative to consider the concepts of symbolic and literal immortality, which are components of religiosity and may also exist on a spectrum, each exerting its unique influence on DA. As depicted in Figure 13, non-religious individuals might strongly embrace symbolic immortality, believing that their legacy will endure beyond their physical existence, thereby acting as a defence against DA (Greenberg et al., 2014). Conversely, those with a weaker sense of symbolic immortality may still experience reduced DA if their conviction in literal immortality, such as the possibility of an afterlife or eternal existence after death, remains robust. When individuals hold firm beliefs in literal immortality, it can assuage the existential apprehension linked to death by providing reassurance at the thought of continued existence. Likewise, even moderately religious individuals with a Quest orientation and religious doubt may experience diminished DA if they maintain a robust sense of symbolic immortality, which serves as a protective factor against existential anxieties. Thus, the connection between religiosity and DA unfolds as a multifaceted and intricate interplay of these factors.

### Consistent DA and religiosity across age groups

We failed to reject *H*_2_, which states that young and middle-aged adults would exhibit higher DA scores compared to older adults. While our study found that older adults tend to have lower DA than young and middle-aged adults, this difference was not statistically significant. Instead, our results show that DA levels are relatively consistent across the three age groups in our sample. This contradicts previous research, which shows that young and middle-aged adults tend to report greater DA than older adults (Russac et al., 2007; Chopik, 2016). However, it is important to note that our sample contained a small proportion of older adults (12.7%), with the majority being young and middle-aged adults. The proportion of older adults in our study also fell below the national average of 18.4% (Chin, 2022).

The underrepresentation of older adults in our study may be due to our stringent inclusion criteria, which required participants to have no severe psychiatric conditions or recent episodes of mental health distress in order to safeguard their well-being. While we made concerted efforts to increase the participation of older adults, including distributing flyers and collaborating with active aging centers, these endeavors had limited success. Notably, the active aging centers primarily serve vulnerable seniors in need of additional social support (Agency for Integrated Care, 2023), and hence, may not have met our study’s inclusion criteria. Additionally, limited access to online surveys (the primary mode of data collection), potential language barriers, and the reluctance to engage in a study involving the taboo topic of death in Singapore, especially among older individuals, further compounded the issue (Lee, 2009; Ang et al., 2016). Ultimately, the challenge of recruiting older adults for a taboo topic while maintaining stringent inclusion criteria may have hindered our ability to identify significant age-related differences in DA.

We failed to reject *H_3_*, which predicted that older adults would report higher religiosity scores compared to middle-aged and young adults. Our analysis did not reveal a significant difference in religiosity across the three age groups, contrary to prior findings (Bengtson et al., 2015) and the gerotranscendence theory (Tornstam, 1997), which suggests that older adults tend to turn toward transcendental engagements and spiritual fulfilment in their later years. Again, the limited representation of older adults in our sample compared to young and middle-aged adults may have influenced these results. Beyond that, however, the evolving religious landscape in Singapore which is marked by a growing trend of secularization could have played a more significant role. Between 2011 and 2021, the proportion of the Singapore population identifying as non-religious increased from 17 to 20% (Department of Statistics Singapore, 2011; Department of Statistics Singapore, 2021a). This modest yet noteworthy shift implies a general trend of decreasing religiosity within this population. Considering that our study took place in 2023, if the trend holds, we could anticipate a further reduction in religiosity among our participants, which may account for the homogenous levels of religiosity observed between older and younger adults in our study.

#### Contributing factors and psychosocial development

As our study found that age does not significantly influence DA or religiosity, we suggest that these multifaceted constructs may instead be influenced by factors beyond age, such as individual characteristics, life experiences, health conditions, and the meaning derived from literal or symbolic immortality across the lifespan. Erikson’s (1980) theory of psychosocial development offers a valuable lens. It posits that individuals navigate eight stages, each presenting unique challenges and opportunities for growth. Importantly, these stages are not age-bound; individuals of any age can face difficulties progressing the stage they are in, thereby impacting their existential views. For example, an individual in their 60s may still grapple with identity formation and experience existential questioning similar to someone in their 20s should the stage remain unresolved. Distinct from age, societal upheavals or personal traumas can also prompt existential reflection and influence beliefs about life’s meaning and death.

Health issues are another factor that directly impacts DA. Individuals of all ages can develop terminal or chronic illnesses, and the resulting psychological and physical pain can overshadow their overall well-being and lead to reduced levels of DA, as suggested by DAT (Ellis et al., 2013) and supported by previous research (Cicirelli, 2003; Wysokiński et al., 2019). Additionally, the phenomenon of experiencing negative life transitions, which may drive older adults to seek solace in religion (Bengtson et al., 2015), is not confined solely to this age group. Young and middle-aged adults may also turn to religion as a source of comfort and support when confronted with life-altering challenges. This intricate interplay between life events, health conditions, and an individual’s spiritual or religious beliefs exert a significant influence on both DA and religiosity. The absence of age-related differences in DA and religiosity in our study underscores the importance of considering the broader context of an individual’s life and experiences, including their stage of psychosocial development as outlined by Erikson (1980), when evaluating these multifaceted constructs.

### Cultural perspectives on gender, religiosity and DA

We found support for *H*_4_, which posited that women would report greater religiosity scores compared to men. Our findings are consistent with prior research (Francis and Penny, 2014; Hoffmann, 2019; Bryukhanov and Fedotenkov, 2023). This inclination could be linked to the observation that women generally exhibit lower risk tolerance than men (Miller and Hoffmann, 1995). Their increased cautiousness may predispose them toward greater religiosity as a means to alleviate concerns about facing potential consequences in the afterlife for not adhering to religious beliefs.

We also found support for *H*_5_, which proposed that women would report greater DA scores compared to men. This implies that, although the women in our study might indeed exhibit greater religiosity, their religious beliefs may not necessarily function as a means to cope with DA. The possibility exists that their religiosity leans primarily toward extrinsic or Quest orientations, or they may not derive a profound sense of symbolic immortality, or both (see Figure 13). As previously mentioned, having an extrinsic or Quest religious orientation is often associated with heightened DA, and a diminished sense of symbolic immortality does not provide effective protection against DA. Our results challenge the simplified notion that religiosity uniformly functions as a universal coping mechanism for DA. Religiosity’s multifaceted nature, encompassing literal and symbolic immortality, and various religious orientations, introduces significant complexity to the associations between religiosity, gender, and the experience of DA.

The gender gap, where women tend to experience higher DA compared to men, is observed across various cultural contexts, highlighting the interplay of cultural and social factors. Research conducted in Asian and Middle-Eastern cultures, characterized by rigid gender roles and limited mobility for women, suggests that men often display a stronger sense of symbolic immortality due to their greater autonomy and social status (Saleem and Saleem, 2019; Adelirad et al., 2021). This heightened sense of symbolic immortality may empower men to pursue diverse careers, engage in public life, and assert personal autonomy–opportunities that women may not enjoy to the same extent. Consequently, women may perceive their lives as more constrained, hindering their personal growth and self-expression. Such constraints can diminish their sense of symbolic immortality, leading to elevated DA as women may feel their societal roles limit the significance of their lives. In these cultural contexts, the experience of heightened DA in women may stem from a reduced sense of symbolic immortality.

Research conducted in Western cultures, where gender roles are predominantly flexible and women have greater autonomy, indicates that women also experience higher levels of DA. This phenomenon is attributed to concerns about dependency in later years, feelings of undesirability, and ageist stereotypes (Dattel and Neimeyer, 1990; Schafer and Shippee, 2010). Specifically, it is suggested that women in Western cultures often harbor negative expectations about aging, driven by a lack of confidence in their cognitive abilities. This pessimistic outlook may heighten women’s apprehension about the decline of their mental acuity and memory as they age (Schafer and Shippee, 2010). Despite the relative freedom and autonomy of women in Western societies, they face unique societal pressures and expectations that contribute to increased DA, particularly concerning cognitive aging.

Singapore uniquely blends Asian traditions and Western influences, earning it the reputation of “East meets West” (Li et al., 2008). Its diverse population primarily comprises four main racial groups: Chinese, Malays, Indians, and Eurasians. Despite the establishment of gender equity under the law, ethnic-cultural norms continue to influence gender roles in Singapore. Take, for instance, women of East Asian heritage in Singapore who may contend with Confucian expectations concerning caregiving responsibilities. These expectations place the onus of maintaining family harmony and upholding traditional domestic ideals squarely on women (Fang, 2021). In Malay culture, there may exist an expectation for men to assume the role of being the head of the household, while women typically take on subservient roles (Abdullah et al., 2008). Similarly, within Indian culture, women have traditionally been assigned the role of homemakers, while men often wield decision-making authority in family matters, encompassing financial decisions and matters concerning their children’s education and marriage (Sivakumar and Manimekalai, 2021). These deeply entrenched expectations may erode symbolic immortality and intersect with Westernized values that prioritize individualism and independence, creating a dynamic tension that shapes how women navigate their societal roles. Therefore, a combination of negative perceptions regarding cognitive decline, ageist stereotypes, and the weight of cultural expectations may collectively contribute to the heightened levels of DA observed in the women within our sample.

### Implications

As our study was of an exploratory nature, it carries two main implications. Foremost, it highlights that DA is prevalent among highly and moderately religious people. This observation strongly implies that DA is likely to be prevalent in Singapore, a nation where 80% of the population is religious. Furthermore, our research reveals that DA levels remain consistent across different age groups. This suggests that DA affects people of all ages in Singapore, emphasizing its wide-reaching impact. Despite the burden that DA imposes on mental health, it has received limited attention in Singapore. This gap in understanding and addressing DA is concerning given its widespread prevalence. Our study serves as a vital catalyst for recognizing and addressing the significance of DA in the context of Singapore’s mental health, calling for further research and targeted interventions to support those grappling with this issue.

Accordingly, our study highlights the need for targeted DA interventions that integrate spiritual aspects into therapy, a practice known to be effective in aiding individuals with psychological disorders, including DA (Friedrich-Killinger, 2020). This integration is vital for culturally competent care among clinicians and to effectively assist those struggling with DA. Culturally competent care with spiritual considerations incorporated may include: assessing spiritual and cultural beliefs and practices through open-ended questions; respecting the diverse spiritual and cultural beliefs and practices of participants; and aligning therapy goals with participants’ spiritual and cultural values and principles, such as achieving peace and acceptance in the face of death (Friedrich-Killinger, 2020). Namely, our study shows that women, especially, may benefit from such interventions as they have greater religiosity and heightened DA.

### Limitations

Our study has several limitations. Despite efforts to ensure robustness, some variables did not adhere to normality assumptions, necessitating the use of non-parametric tests. This limitation may have implications for the generalizability of the findings, particularly in contexts where the assumptions of parametric tests may not hold. Future studies should explore alternative statistical approaches or consider strategies to address non-normal distributions to enhance the reliability and generalizability of the results.

A significant constraint lies in our relatively small sample size which may not be fully representative of the broader population. This limitation could be attributed, in part, to the cultural taboos surrounding discussions of death within Singapore society. Such taboos may have fostered reluctance among individuals to engage with the topic of death, potentially discouraging some from participating in our study. This may also explain why we had difficulty recruiting older adults despite reaching out to active aging centers.

Another limitation pertains to our categorization of religiosity and age into groups rather than employing continuous variables. Grouping data can diminish the sensitivity of statistical tests, making it more challenging to discern subtle differences between categories. Consequently, we may not have fully captured the nuanced effects related to these variables. However, it is important to note that our grouping was not arbitrary; we have based it on well-established psychometric scales with prior validation in research. This intentional approach aimed to ensure homogeneity within the defined groups, thereby enhancing our ability to detect meaningful differences and trends in our data.

### Future directions

To address these limitations, future research should employ larger, more diverse samples and incorporate data collection on religious orientation, given that different religious orientations influence DA. Moreover, the observed distinctions in religious and cultural customs between our study in Singapore and prior research conducted in societies with Western religious heritages underscore the necessity for additional follow-up studies. These studies should ambitiously recruit participants from numerous countries and cultures worldwide to enable thorough and accurate examinations, as well as cross-national and cross-cultural comparisons.

It may also be judicious to establish robust safeguards so that sampling DA in vulnerable populations, such as those with psychological conditions, would be made ethically possible. As DA is a transdiagnostic construct and may be present in those with psychological disorders, it is essential to ensure that these populations are well-represented in research studies.

## Conclusion

Our study addressed the understudied nature of DA in Singapore despite its significant impact on mental health. Additionally, research on the DA-religiosity relationship in multicultural settings like Singapore is limited, while research on the curvilinear relationship, which posits that moderately religious people have higher DA than highly religious and non-religious individuals, has significant limitations. To address these gaps, we investigated the curvilinear relationship in Singapore while examining the effects of age and gender. Our results revealed a rising curve pattern instead, where highly and moderately religious people have higher DA than non-religious people. This finding highlights the multifaceted nature of religiosity and suggests that its role as a coping mechanism for DA is intricate, depending on specific religious orientations and an individual’s sense of both symbolic and literal aspects of immortality. We also found that religiosity did not differ significantly across age groups, but women were more religious than men. Similarly, DA did not vary significantly with age, but women had higher levels than men. These findings emphasize the need for targeted DA interventions in Singapore, given the prevalence of religiosity and the higher levels of DA among women. We recommend integrating spiritual aspects into therapy to provide culturally competent care for people with DA.

## Data availability statement

The original contributions presented in the study are included in the article/Supplementary material, further inquiries can be directed to the corresponding author.

## Ethics statement

The studies involving humans were approved by Psychology Programme Ethics Review Committee at the Singapore University of Social Sciences (250523). The studies were conducted in accordance with the local legislation and institutional requirements. The participants provided their written informed consent to participate in this study.

## Author contributions

RB: Conceptualization, Data curation, Formal analysis, Investigation, Methodology, Project administration, Resources, Software, Validation, Visualization, Writing – original draft, Writing – review & editing. KG: Conceptualization, Supervision, Writing – review & editing.
