# Correction: Death anxiety and religiosity in a multicultural sample: a pilot study examining curvilinearity, age and gender in Singapore

**DOI:** 10.3389/fpsyg.2025.1729710

**Published:** 2025-11-13

**Authors:** Radiah Maria Belak, Kay Hee Goh

**Affiliations:** Singapore University of Social Sciences, Singapore, Singapore

**Keywords:** death anxiety, spirituality, religiosity, existentialism, death and dying, transdiagnostic construct

A correction has been made to text in the **Methodology** section to remove personally identifying information.

The original version of this article has been updated.

