# Supplementary material for: Death anxiety and religiosity in a multicultural sample: a pilot study examining curvilinearity, age and gender in Singapore
Source: Front Psychol. 2024 May 22;15:1398620. doi: 10.3389/fpsyg.2024.1398620 (PMC11165362; doi:10.3389/fpsyg.2024.1398620)

**Appendix A**

**Participant Information Sheet**


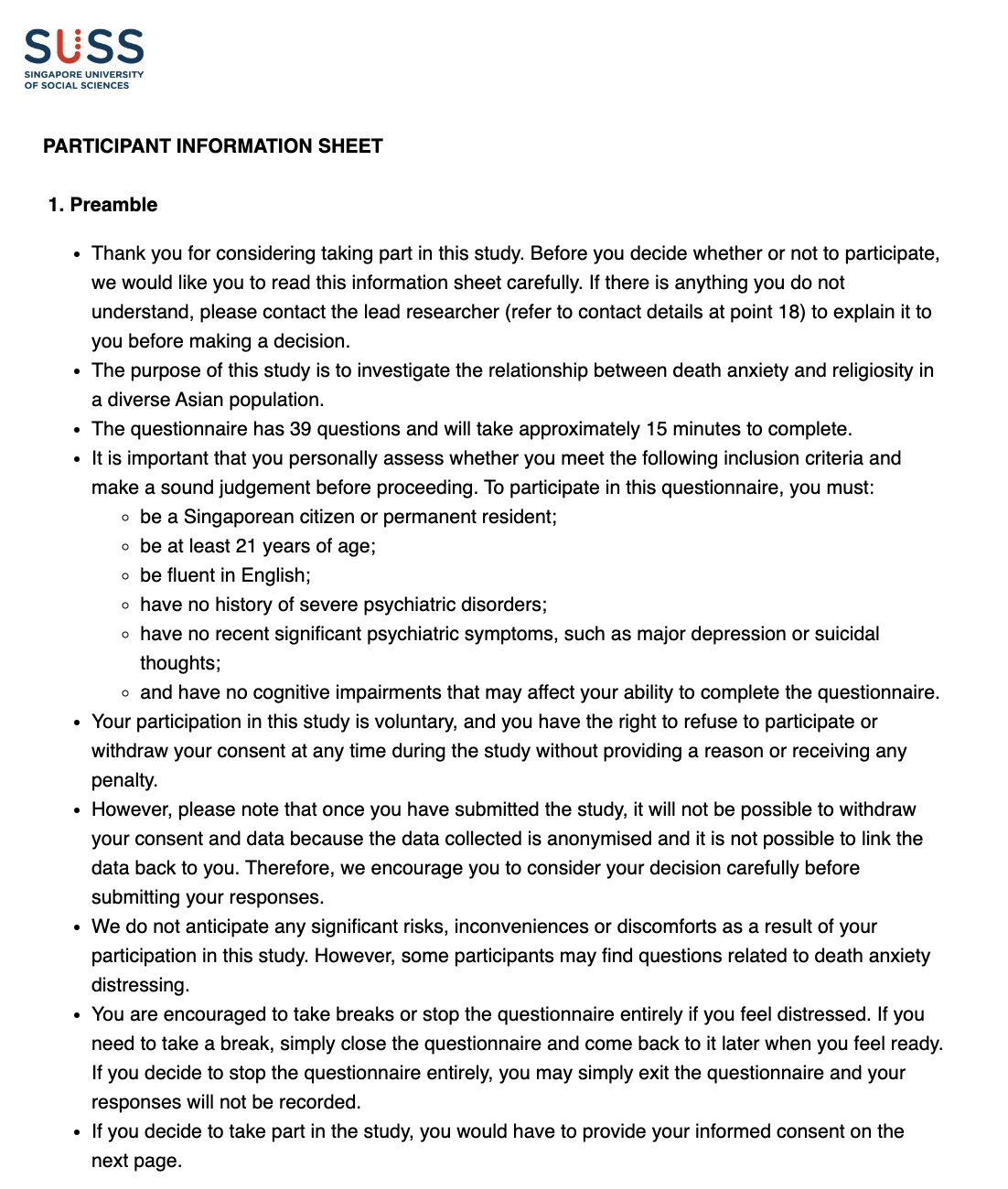


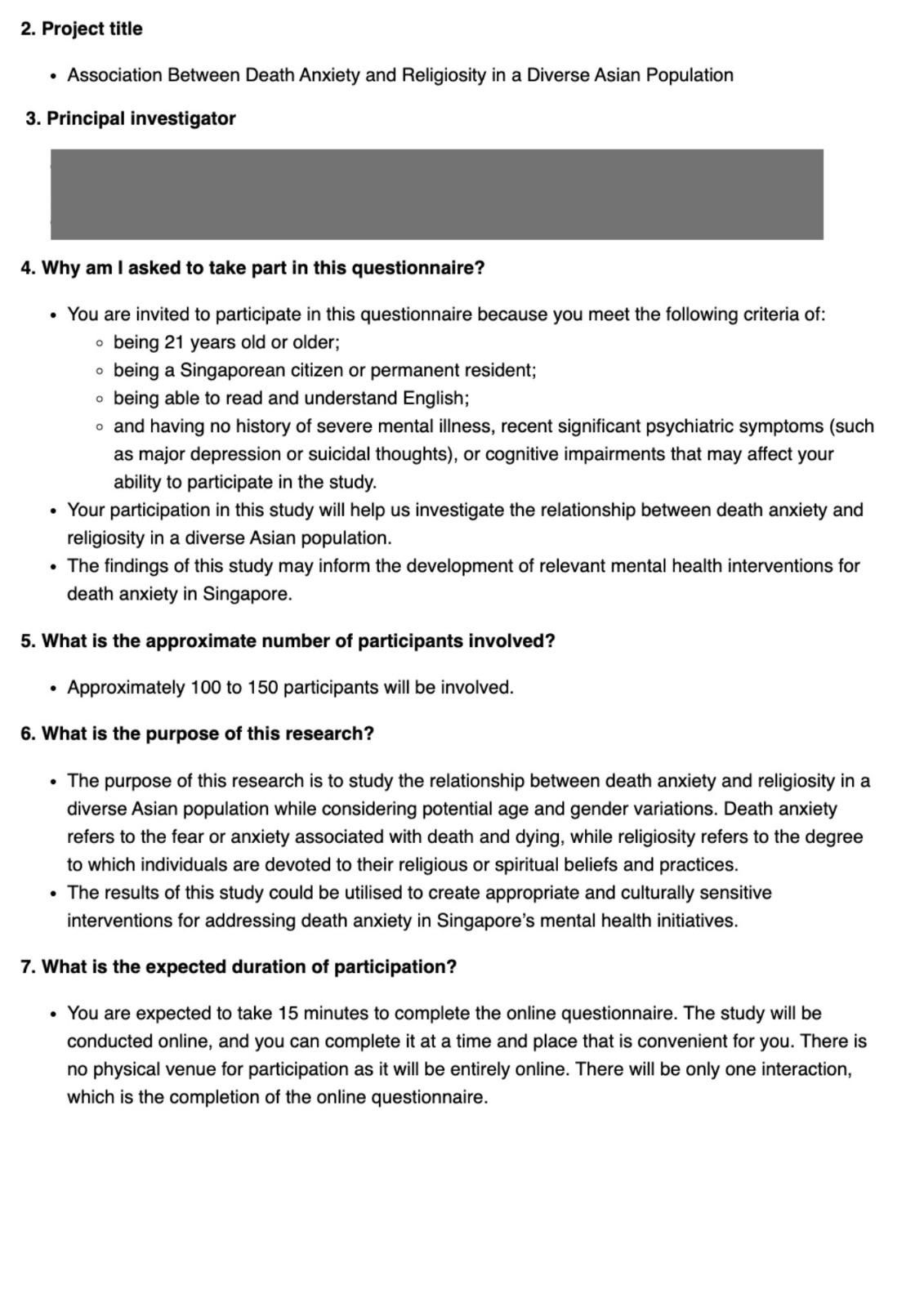


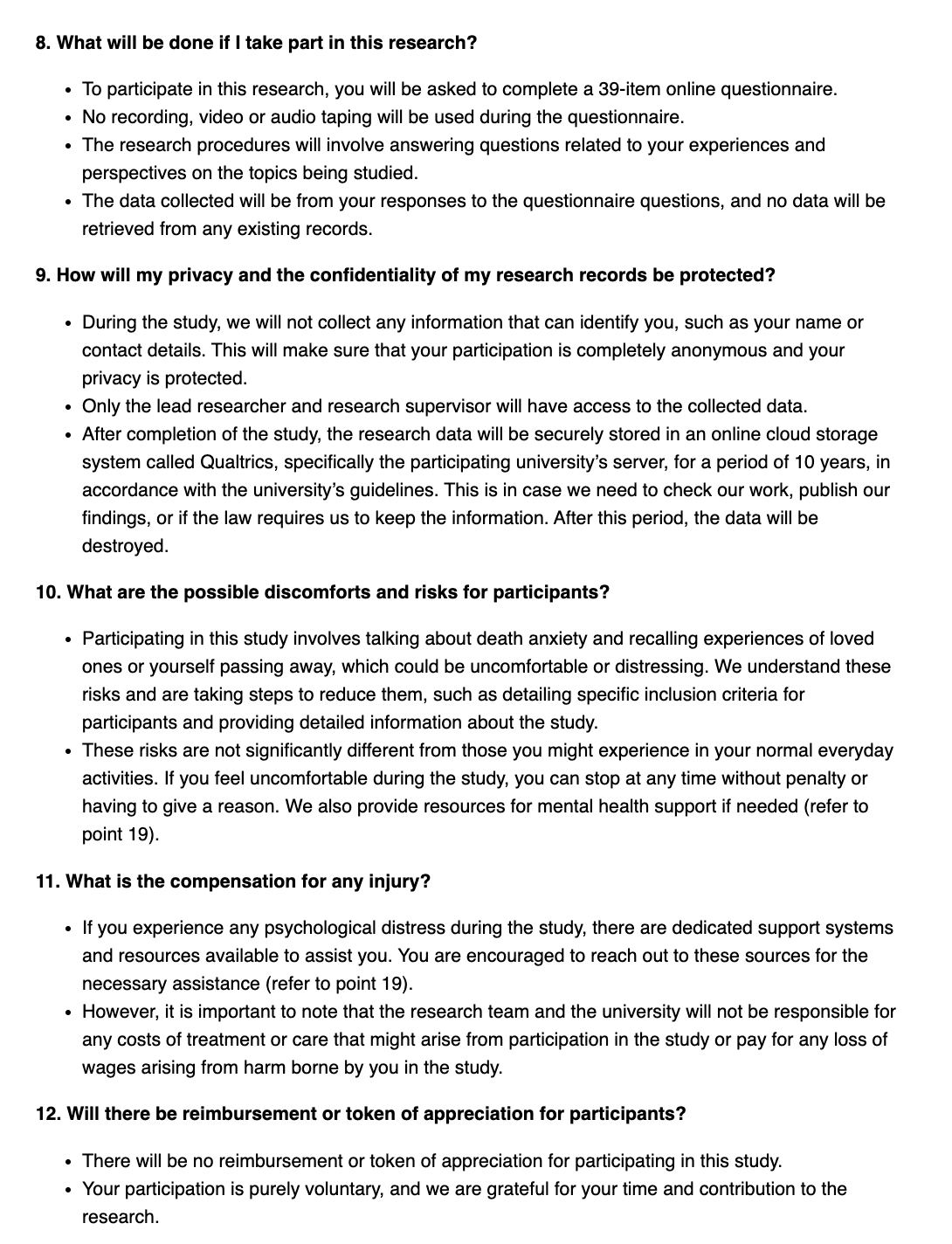


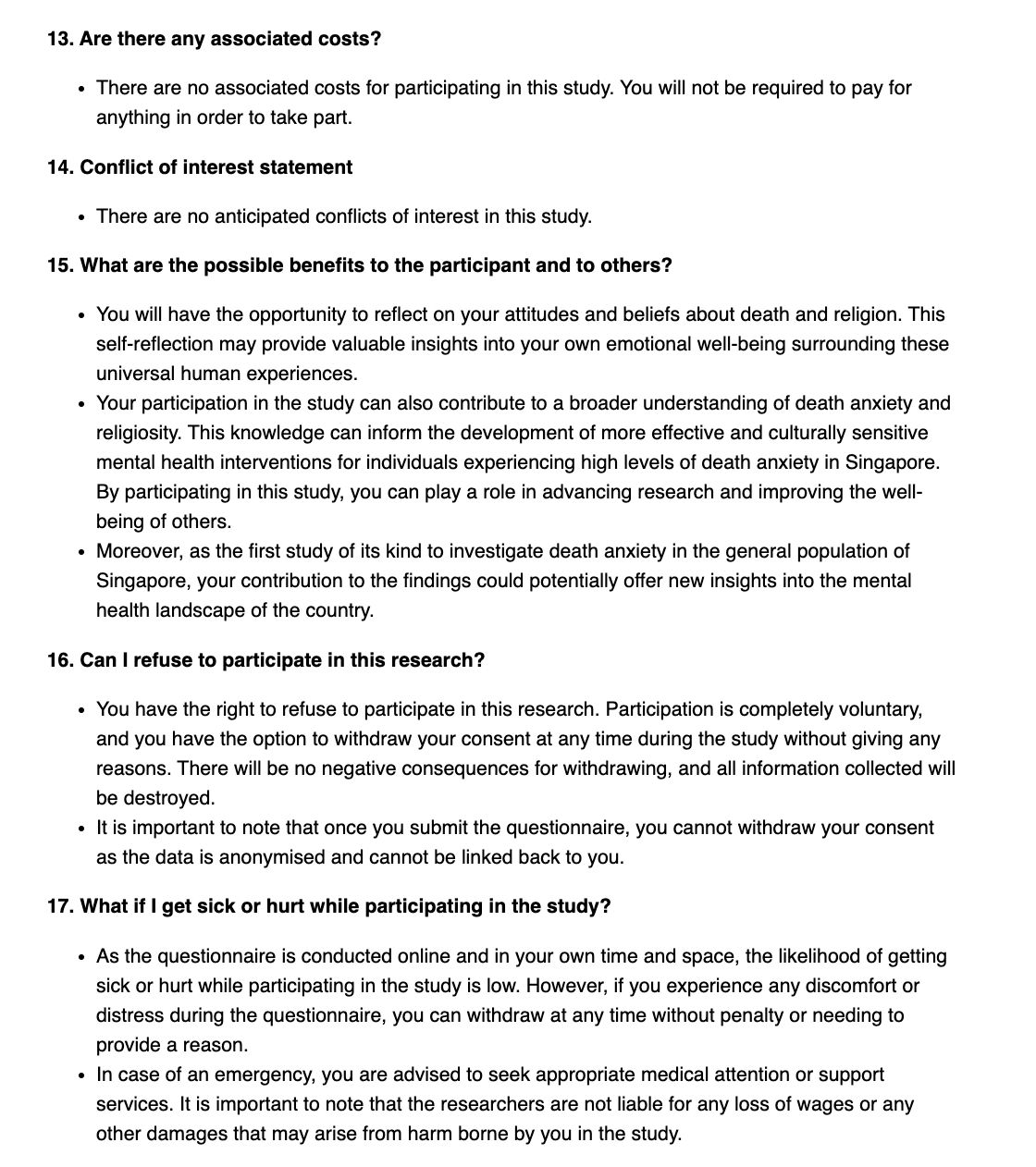


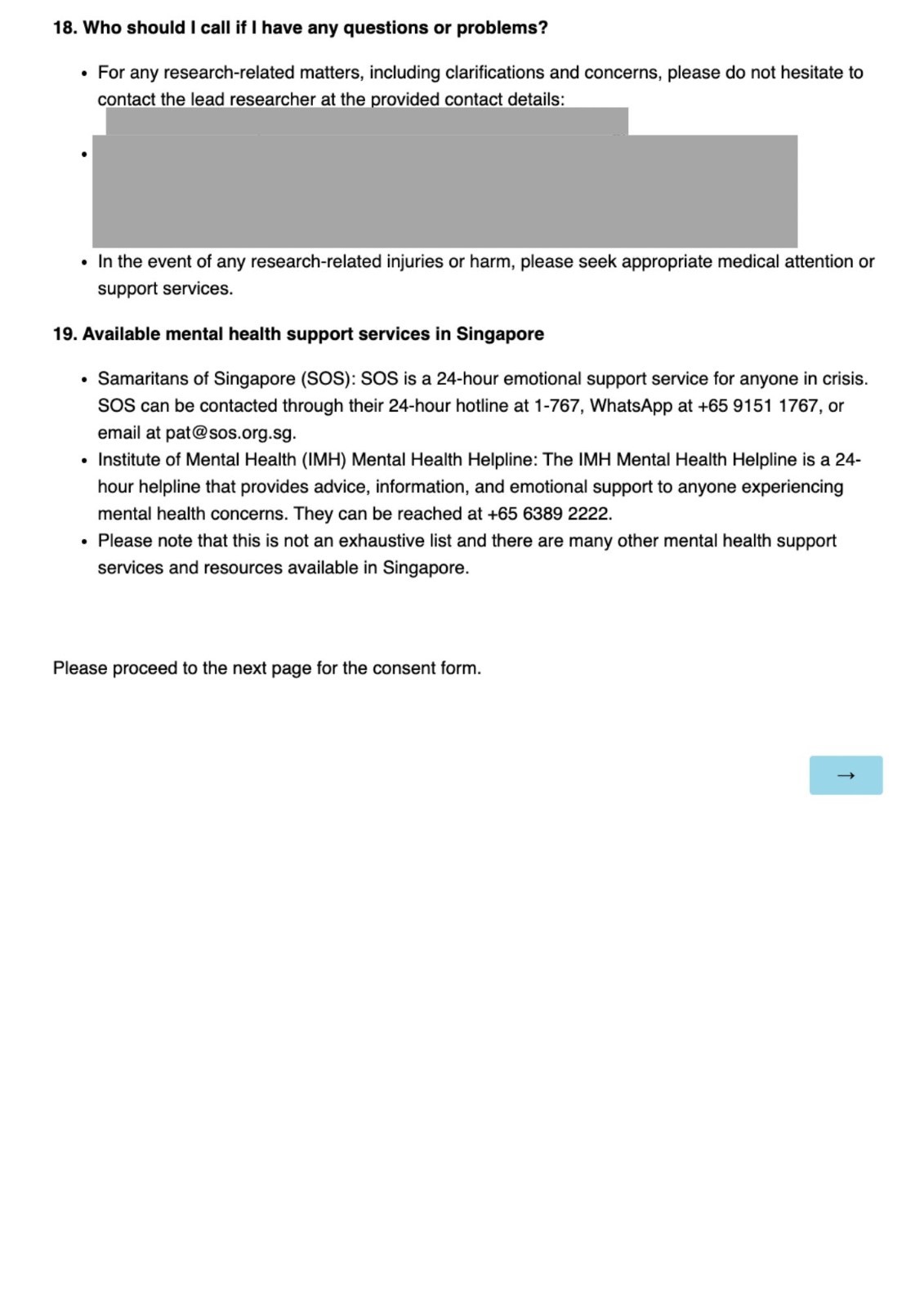


**Appendix B**

**Informed Consent Form**
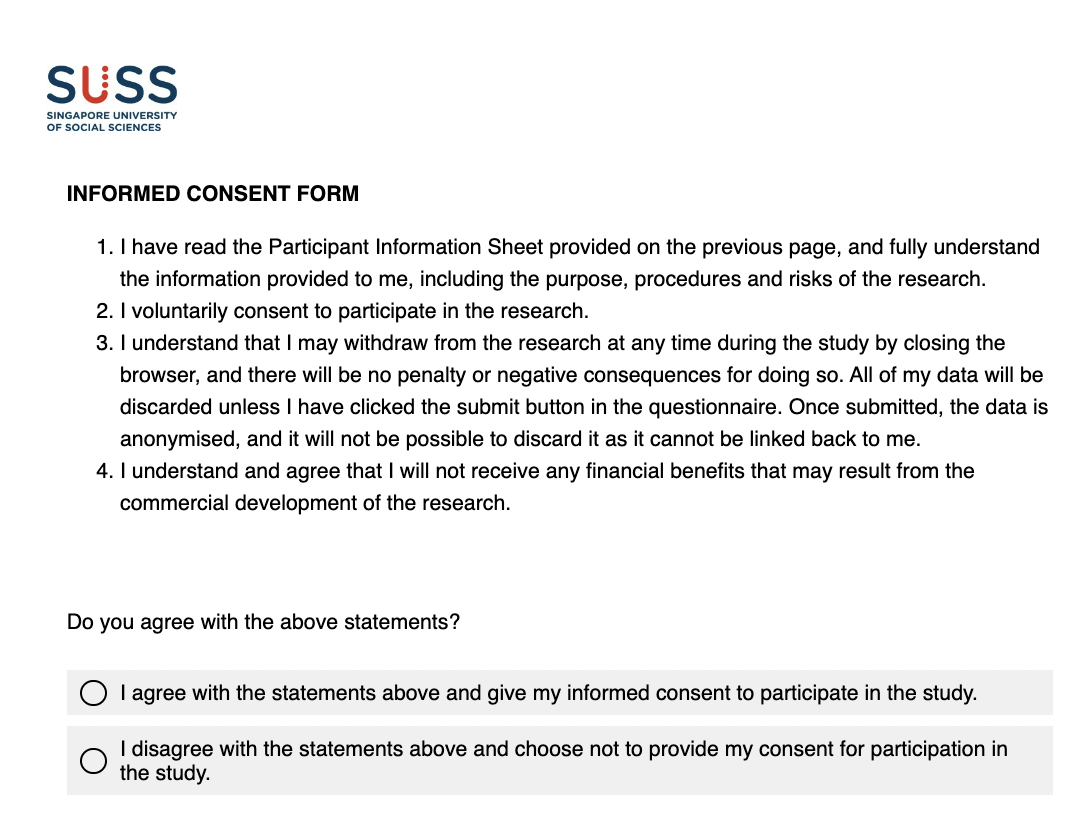


**Appendix C**

**Survey Demographic Questions**

1. What is your age group?
   - 21 to 34
   - 35 to 64
   - 65 and above
2. What is your gender?
   - Female
   - Male
   - Others

**Appendix D**

**The Revised Collett-Lester Fear of Death Scale (CLFDS-R; Lester, 1990)**


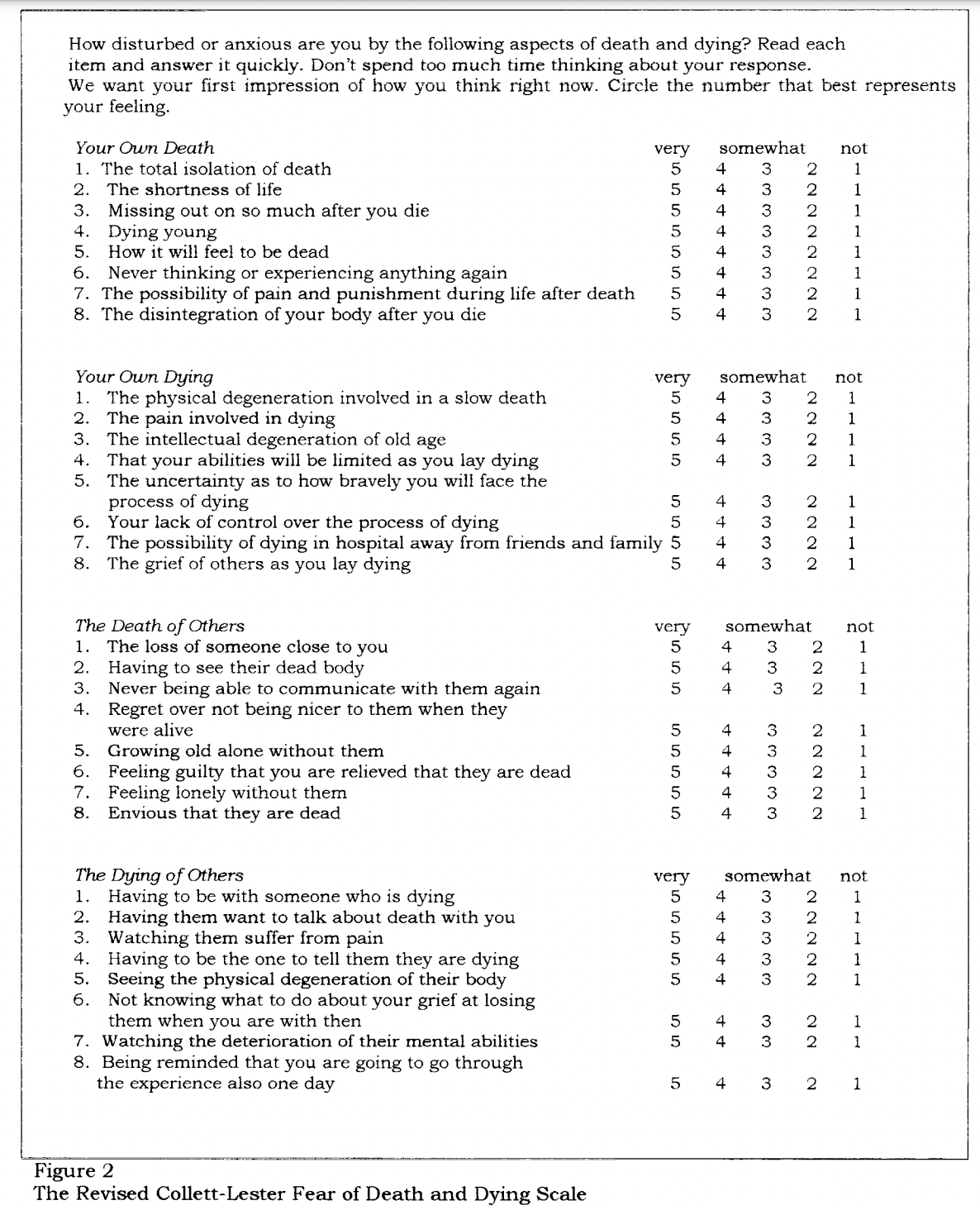


**Appendix E**

**The Centrality of Religiosity Scale (CRSi-7; Huber & Huber, 2012)**


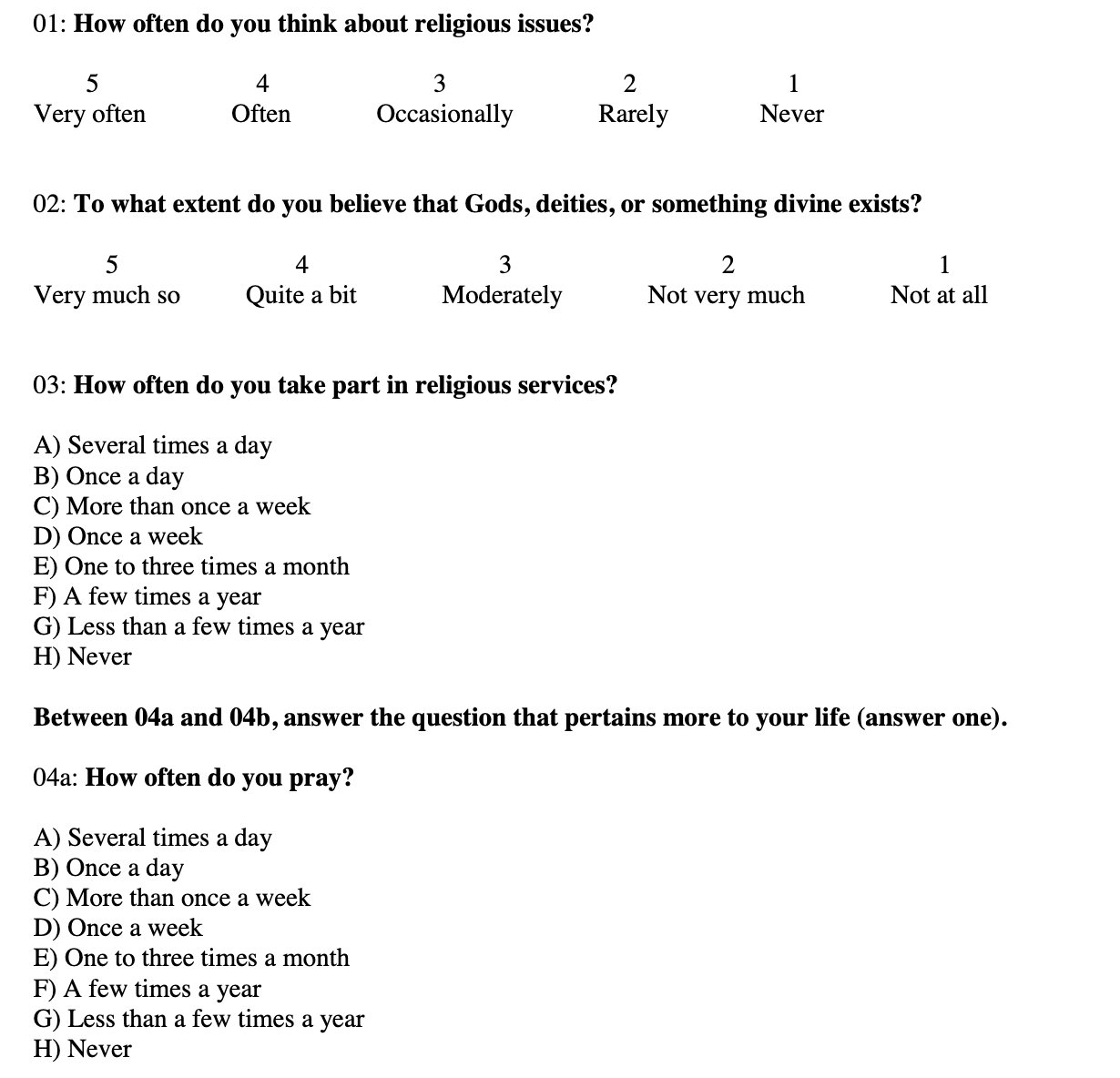


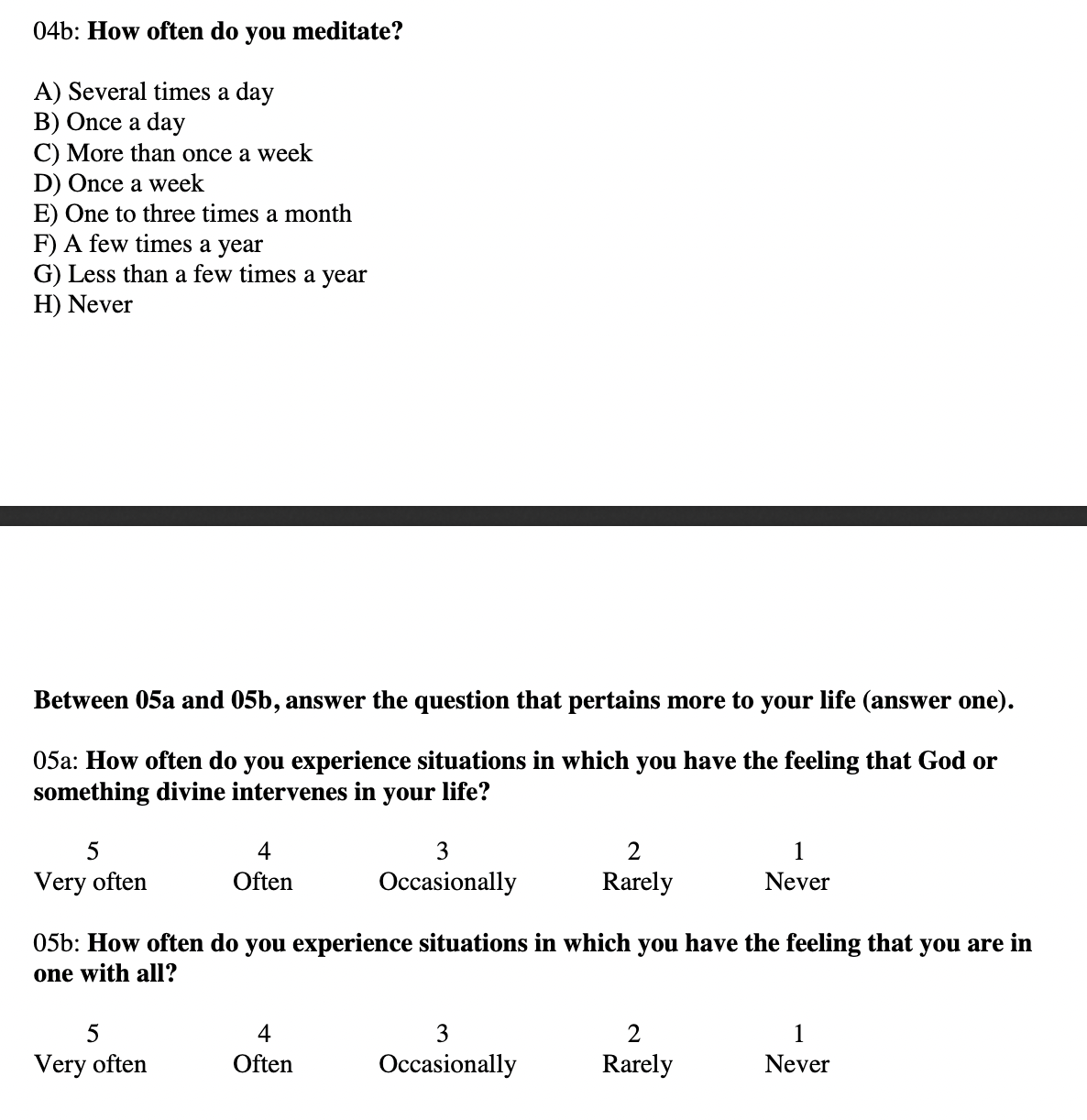


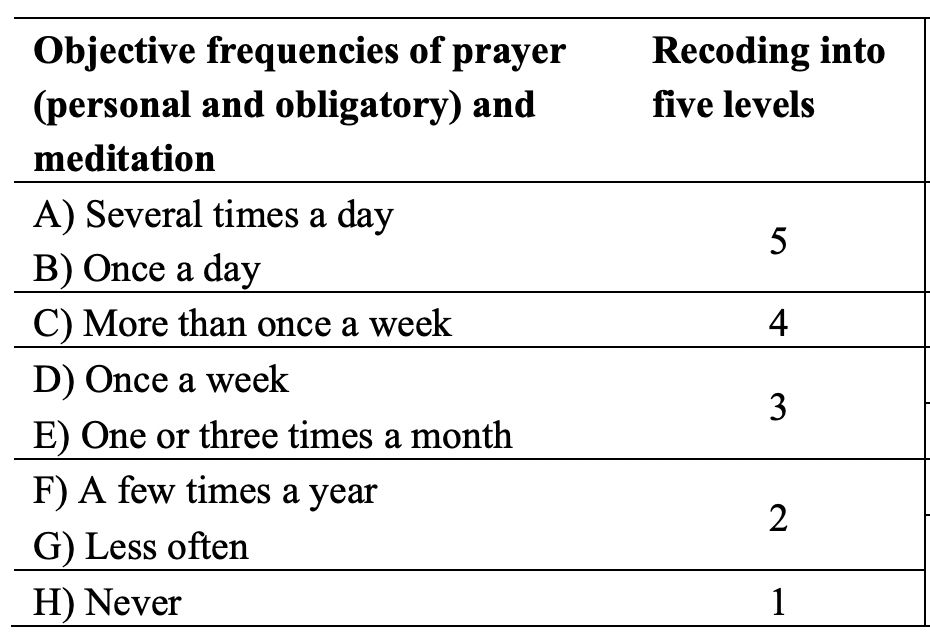


**Appendix F**

**Debriefing Statement**


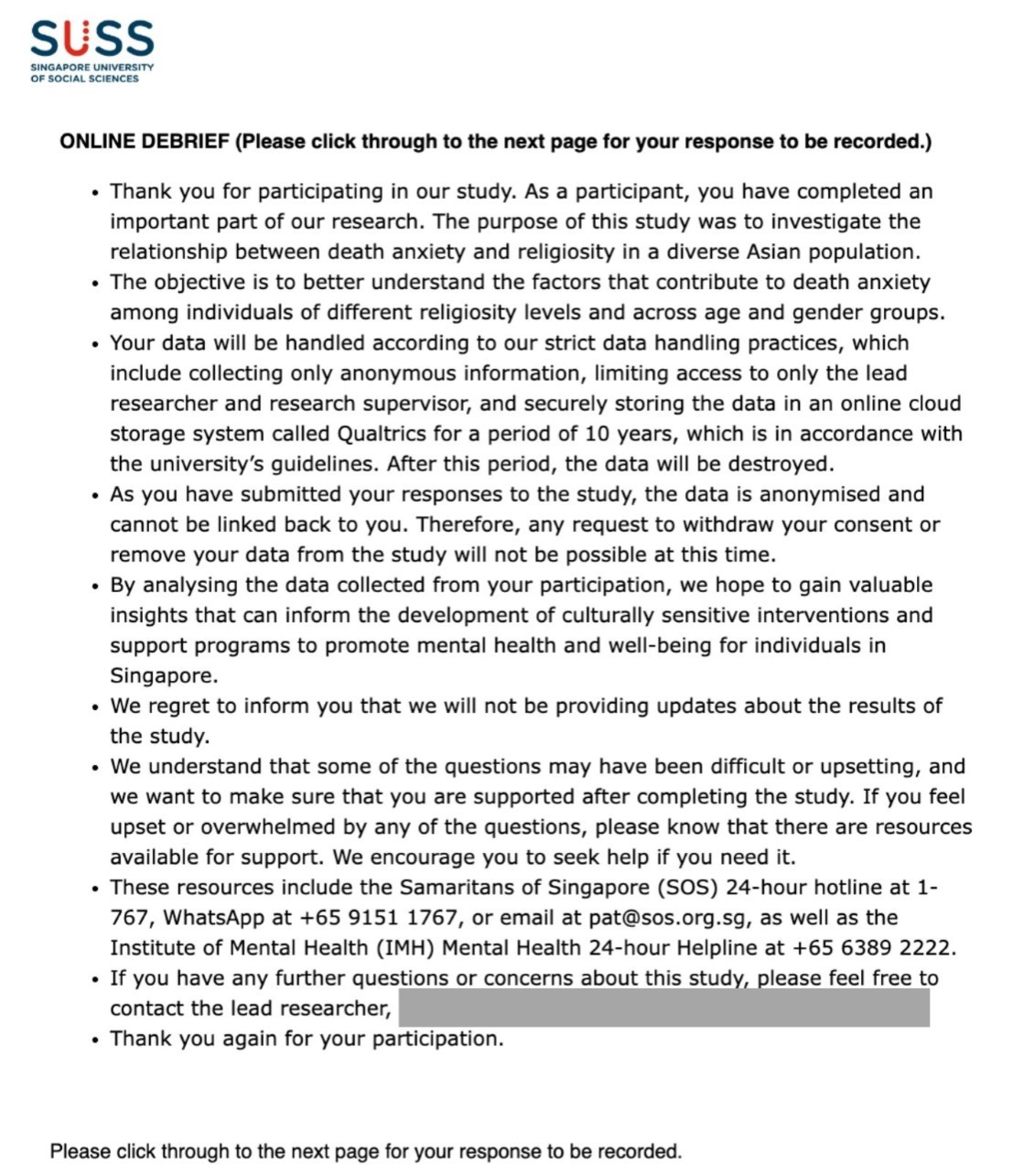

Supplement: Supplementary file 1 [file Data_Sheet_1.docx]
